# The TOR Pathway Is Involved in Adventitious Root Formation in *Arabidopsis* and Potato

**DOI:** 10.3389/fpls.2017.00784

**Published:** 2017-05-12

**Authors:** Kexuan Deng, Pan Dong, Wanjing Wang, Li Feng, Fangjie Xiong, Kai Wang, Shumin Zhang, Shun Feng, Bangjun Wang, Jiankui Zhang, Maozhi Ren

**Affiliations:** ^1^School of Life Sciences, Chongqing UniversityChongqing, China; ^2^College of Agronomy and Biotechnology, Southwest UniversityChongqing, China; ^3^Key Laboratory of Eco-Environments in Three Gorges Reservoir Region, Ministry of Education, College of Life Sciences, Southwest UniversityChongqing, China

**Keywords:** potato, target of rapamycin, adventitious root formation, auxin, *AtTIR1*

## Abstract

In the agriculture industry, adventitious root formation is a core issue of plants asexual propagation. However, the underlying molecular mechanism of adventitious root formation is far beyond understanding. In present study we found that target of rapamycin (TOR) signaling plays a key role in adventitious root formation in potato and *Arabidopsis*. The core components of TOR complex including TOR, RAPTOR, and LST8 are highly conserved in potato, but the seedlings of potato are insensitive to rapamycin, implying FK506 Binding Protein 12 KD (FKBP12) lost the function to bridge the interaction of rapamycin and TOR in potato. To dissect TOR signaling in potato, the rapamycin hypersensitive potato plants (BP12-OE) were engineered by introducing yeast *FKBP12* (*ScFKBP12*) into potato. We found that rapamycin can significantly attenuate the capability of adventitious root formation in BP12-OE potatoes. KU63794 (KU, an active-site TOR inhibitor) combined with rapamycin can more significantly suppress adventitious root formation of BP12-OE potato than the single treatments, such as KU63794 or rapamycin, indicating its synergistic inhibitory effects on potato adventitious root formation. Furthermore, RNA-seq data showed that many genes associated with auxin signaling pathway were altered when BP12-OE potato seedlings were treated with rapamycin + KU, suggesting that TOR may play a major role in adventitious root formation via auxin signaling. The auxin receptor mutant *tir1* was sensitive to TOR inhibitors and the double and quadruple mutants including *tir1afb2, tir1afb3*, and *tir1afb1afb2afb3* displayed more sensitive to asTORis than single mutant *tir1*. Consistently, overexpression of *AtTIR1* in *Arabidopsis* and potato can partially overcome the inhibitory effect of asTORis and promote adventitious root formation under asTORis treatments. These observations suggest that TOR signaling regulates adventitious root formation by mediating auxin signaling in *Arabidopsis* and potato.

## Introduction

During the growth and development of plants, root systems play fundamental roles in absorbing water and mineral nutrition, anchoring plant, and synthesis of various growth factors such as hormone to regulate plant growth and development(Torrey and Phillips, 1974; Russell, 1977; Ljung et al., 2005). As a very specific kind of root, adventitious roots originated from stems or leaf tissues are induced by many environmental and physiological stresses for expanding the absorbing areas or enhancing the resistance to adversity (Klerk et al., 1999; Chu et al., 2008; Li et al., 2009; Bellini et al., 2014). Recently, adventitious root formation has become a core issue both on the theoretical aspect of plant development, organogenesis and on the practical side of plant asexual propagation (Thorpe et al., 1991; Klerk et al., 1999; Abarca and Díazsala, 2009; Li et al., 2009; Amissah et al., 2013; Bellini et al., 2014; Liu et al., 2014; Verstraeten et al., 2014; Hu and Xu, 2016). In the past few years, the formation of adventitious roots had been widely exploited in horticultural and forest species and some progresses had been made in the underlying mechanism of adventitious root formation (Klerk et al., 1999; Abarca and Díazsala, 2009; Li et al., 2009; Bellini et al., 2014; Liu et al., 2014; Chen et al., 2016; Hu and Xu, 2016). Potato is the world's major staple food and also will become the fourth largest staple food in China following by wheat, rice, and maize (Xu et al., 2015). On the other hand, adventitious root formation with stems in potato is very fast, and clonal propagation of potato seedlings is efficient in the laboratory. The study of its mechanism in adventitious root formation may resolve the obstacle of asexual propagation in the other species. Additionally, the available whole-genome sequence and efficient and reliable transformation systems of potato allow it to become an important research model for adventitious root formation.

Adventitious root formation is a complex process, controlled by multiple environmental and physiological factors (Li et al., 2009; Bellini et al., 2014). As crucial elements, phytohormones, particular auxin, play an important role in adventitious root formation (Bellini et al., 2014; Liu et al., 2014; Su and Zhang, 2014). In the induction of stem cuttings to generate adventitious roots, auxin was applied usually in horticultural practice (Klerk et al., 1999; Li et al., 2009). The formation of adventitious root primordia, which is a key step in adventitious root formation, begins with the auxin synthesis and accumulation (Sukumar et al., 2013; Liu et al., 2014; Chen et al., 2016; Rovere et al., 2016). With the accomplishment of the auxin accumulation, the expression of some stem cell niche related genes, such as WUSCHEL(WUS)-related homeobox (WOX) protein family, *SHORT ROOT* (*SHR*), and *SCARECROW* (*SCR*) etc. were induced (Liu et al., 2014; Hu and Xu, 2016; Rovere et al., 2016). Besides auxin, multiple environmental factors including nutrient and energy, could also affect adventitious root formation in plants (Tyburski and Tretyn, 2004; Sorin et al., 2005; Agulló-Antón et al., 2010).

Among all eukaryotes, target of rapamycin (TOR), a conserved serine/threonine protein kinase, integrates energy, nutrient, stress and hormone signaling to promote cell proliferation and growth (Wullschleger et al., 2006; Deprost et al., 2007; Laplante and Sabatini, 2012; Ren et al., 2012; Cornu et al., 2013; Henriques et al., 2014; Xiong and Sheen, 2014, 2015). TOR is composed of five conserved domains from N terminus to C terminus: HEAT repeats (Huntingtin, Elongation factor3, PP2A, and TOR1), FAT (FRAP, ATM, and TRRAP domain), FRB (FKBP-rapamycin binding domain), kinase, and FATC (Carboxy-terminal FAT domain)(Xiong and Sheen, 2014, 2015). Null mutation of *TOR* was embryo lethal in *Arabidopsis*, suggesting TOR signaling is essential for endosperm and embryo development in plants (Menand et al., 2002). Rapamycin, an immunosuppressive agent, can specifically interact with FKBP12 and TOR to form a ternary complex to inactivate the kinase activity of TOR (Chiu et al., 1994; Sabatini et al., 1994; Choi et al., 1996; Laplante and Sabatini, 2012). Based on the classic rapamycin-FKBP12-TOR system, TOR signaling pathway have been extensively studied in yeast and mammals (Virgilio and Loewith, 2006; Laplante and Sabatini, 2012). However, the prevailing land plants were resistant to rapamycin because of changeable plants FKBP12. Recent studies showed that overexpression of yeast *FKBP12* (*ScFKBP12*) or human *FKBP12* (*HsFKBP12*) in *Arabidopsis* could confer rapamycin sensitivity to *Arabidopsis* (Xu et al., 1998; Menand et al., 2002; Mahfouz et al., 2006; Sormani et al., 2007; Leiber et al., 2010; Ren et al., 2012). On the other hand, the second generation inhibitors of mTOR, also called active-site TOR inhibitors (asTORis), were developed for cancer therapy by targeting both TORC1 and TORC2 (Benjamin et al., 2011; Montane and Menand, 2013). AsTORis can specifically bind to kinase domain of TOR and compete with ATP to block TOR kinase activities (García-Martínez et al., 2009; Thoreen et al., 2009; Chresta et al., 2010). In recent studies, asTORis were also used in plants to inhibit TOR kinase activity (Montane and Menand, 2013; Xiong et al., 2013, 2016, 2017; Dong et al., 2015; Li et al., 2015; Deng et al., 2016).

In the mammals, TOR plays major roles in the regeneration of animal tissues, such as neurons, liver, and intestinal regeneration (Palmes et al., 2008; Fouraschen et al., 2013; Legacy et al., 2013; Cho et al., 2014; Maiese, 2014; Guan et al., 2015). Researches in *Arabidopsis, Zea mays, Gossypium hirsutum, Solanum lycopersicum*, and *Chlamydomonas creinhardtii* showed that TOR signaling played fundamental roles in seed germination, root and leaf growth, flowering, senescence, and life-span determination in plants (Crespo et al., 2005; Agredano-Moreno et al., 2007; Song et al., 2007; Ren et al., 2011, 2012; Xiong et al., 2013, 2016; Xiong and Sheen, 2014) but little is known about the role of TOR signaling in adventitious root formation in plants.

In this study, adventitious root formation of transgenic potato BP12-OE lines, an engineered rapamycin sensitive system, was retarded by asTORis, rapamycin and their combination. Transcriptome profiling analysis of BP12-OE17 treated with rapamycin, KU, and their combination suggests that a huge number of genes involved in adventitious root formation were regulated by TOR signaling, including numerous auxin signaling pathway related genes. Furthermore, the auxin receptor mutants (*tir1, tir1afb2, tir1afb3*, and *tir1afb1afb2afb3*) showed higher sensitivity to asTORis in adventitious root formation compared to WT, while overexpression of *AtTIR1* in *Arabidopsis* and potato could partially rescue adventitious root formation under TOR inhibitors treatment. These results suggest that TOR may integrate multiple environmental and endogenous factors to regulate adventitious root formation through modulating auxin signaling.

## Materials and methods

### Plant materials and growth conditions

*Arabidopsis* wild type (Columbia, Col-0) was used in this study. All the sterilized and cleaned seeds were put in 4°C for 2 days for vernalization. The 3–4 weeks old potato wild type (*Desiree*) aseptic seedlings were used in this study. For *Arabidopsis* and potato, all the materials were grown in growth chambers which maintained 22°C at a photoperiod of 16 h light (100-microm /m^2^/s fluorescence bulb light) followed by 8 h dark.

### Fresh weight and root growth measurements

The *Arabidopsis* seeds were sown on half-strength Murashige and Skoog medium (0.5 × MS) (Murashige and Skoog, 1962) with different TOR inhibitors at varying concentrations for the time as described in the results section. The potato explants from 3 to 4 weeks old potato seedlings were subculture into 1 × MS with different TOR inhibitors at different concentrations for the time as indicated in the results section. Then all petri dishes were photographed next to a ruler. The root length and fresh weight were measured using ImageJ software and an electronic balance, respectively. The number of different root types was counted directly. Root hair was observed on the OLYMPUS MVX10 stereoscopic microscopes (Olympus, Japan).

### Gus staining, gus activity test, and western blotting

GUS staining and GUS activity test were performed as described previously, respectively (Menand et al., 2002; Li et al., 2015). Western blotting method was following by Deng et al. (2016).

### AXR3-GUS protein degradation assay

5 days old *HS:: AXR3NT-GUS* transgenic seedlings were incubated in 0.5 × MS liquid medium with TOR inhibitors for 48 h, DMSO was used as control. After that seedlings were heat shocked for 2 h at 37°C, and then supplied with NAA (10 μM) for 60 min at 22°C. Finally, the seedlings were stained 6 h for GUS staining.

### RNA extraction and cDNA library construction

Potato stem cuttings growing on the MS media for 48 h with different TOR inhibitors (rapamycin, KU, and their combination) and DMSO were collected and frozen in liquid nitrogen. Total RNA extraction used the RNAprep Pure Plant Kit (TianGen Biotech). RNA degradation and contamination were monitored on 1% agarose gels. RNA purity, concentration and integrity were measured by a NanoPhotometer spectrophotometer (IMPLEN, CA, USA), Qubit RNA Assay Kit in Qubit 2.0 Flurometer (Life Technologies, CA, USA) and the RNA Nano 6000 Assay Kit of the Bioanalyzer 2100 system (Agilent Technologies, CA, USA), respectively. mRNA was purified from about 3 μg total RNA using poly-T oligo-attached magnetic beads. First strand cDNA and subsequent second strand cDNA synthesis were performed by using random hexamer primer. Sequencing adaptors were ligated to the fragments, which were enriched by PCR amplification. At last, PCR products were purified (AMPure XP system) and library quality was assessed on the Agilent Bioanalyzer 2100 system.

### Illumina sequencing and data analysis

Construction of RNA-Seq libraries was performed on an Illumina Hiseq platform and 125/150 bp paired-end reads were generated. After removing the reads containing adapter, poly-N and low quality reads from raw data, the high quality clean reads were obtained. Paired-end clean reads were aligned to the reference genome using TopHat v2.0.12. The reads numbers mapped to each gene were quantified using HTSeq v0.6.1. FPKM (expected number of Fragments Per Kilobase of transcript sequence per Millions base pairs sequenced) of each gene was calculated based on the length of the gene and reads count mapped to this gene. Differential expression analysis between the treatment of TOR inhibitor and DMSO was performed using DESeq R package (1.18.0). Genes with an adjusted *P* < 0.05 found by DESeq were assigned as differentially expressed genes (DEGs). The DEGs were mapped to GO terms in the GO database (http://www.geneontology.org/) to calculate gene numbers in every term. KEGG (http://www.genome.jp/kegg/) is used to perform pathway analysis of DEGs. Statistical enrichment of DEGs in GO terms and KEGG pathway were implemented by the GOseq R package and KOBAS software, respectively. Significantly enriched GO terms and KEGG pathway (corrected *p* < 0.05) were identified based on a hypergeometric test.

### Quantitative real-time PCR (qRT-PCR)

*Arabidopsis* samples were prepared as describe elsewhere in the article. For potatoes, the different tissues (leaf, root, shoot, and stem) of the 4 weeks old potato were collected for measuring the expression level of *StTOR, StFBKP12, StRAPTOR*, and *StLST8* by qRT-PCR. Some genes expression was tested by qRT-PCR as well in the potato samples, which were the same as that used for Illumina sequencing. Total RNA was isolated as described above. Approximately 1 μg of total RNA was used for reverse transcription with the PrimeScript RT Kit (Takara Biotech). The qRT-PCR was conducted using the TranStart Top Green qPCR SuperMix kit (Transgen) on a Bio-Rad CFX96 System. The amplification program consisted of the following cycles: 94°C pre-denaturation for 1 min, 40 cycles of 94°C 5 s, and 60°C 30 s. Plant actin was used as constitutive reference. The primers for qRT-PCR were designed using Primer premier 5 software and were listed in Table S1.

### Generation of overexpression constructs and plant transformation

Total RNA was extracted from yeast cells and *Arabidopsis* using Trizol (Invitrogen), following the manufacture's protocol. cDNA was synthesized by using the PrimeScript RT Kit (Takara). The full-length coding sequence of *ScFKBP12* and *AtTIR1* was amplified by TransStar Taq DNA Polymerase (Transgen) using the designed primers (Table S1). A *Not*I site at the 5′ end of forward primer and a *Sbf* I site at the 3′ end of reverse primer were introduced. The remains steps of plasmid construction were performed as described elsewhere (Ren et al., 2012). For the method of *P35S::TIR1-GUS-K303* vector generation was described elsewhere (Li et al., 2015). The potato transgenic method was according to the book “Agrobacterium protocols” (Springer press, Second Edition; Millam, 2007). The floral dipping method was employed for generating transgenic *Arabidopsis* (Zhang et al., 2006).

## Results

### Putative components of TOR pathway in potato

**Figure 1:**
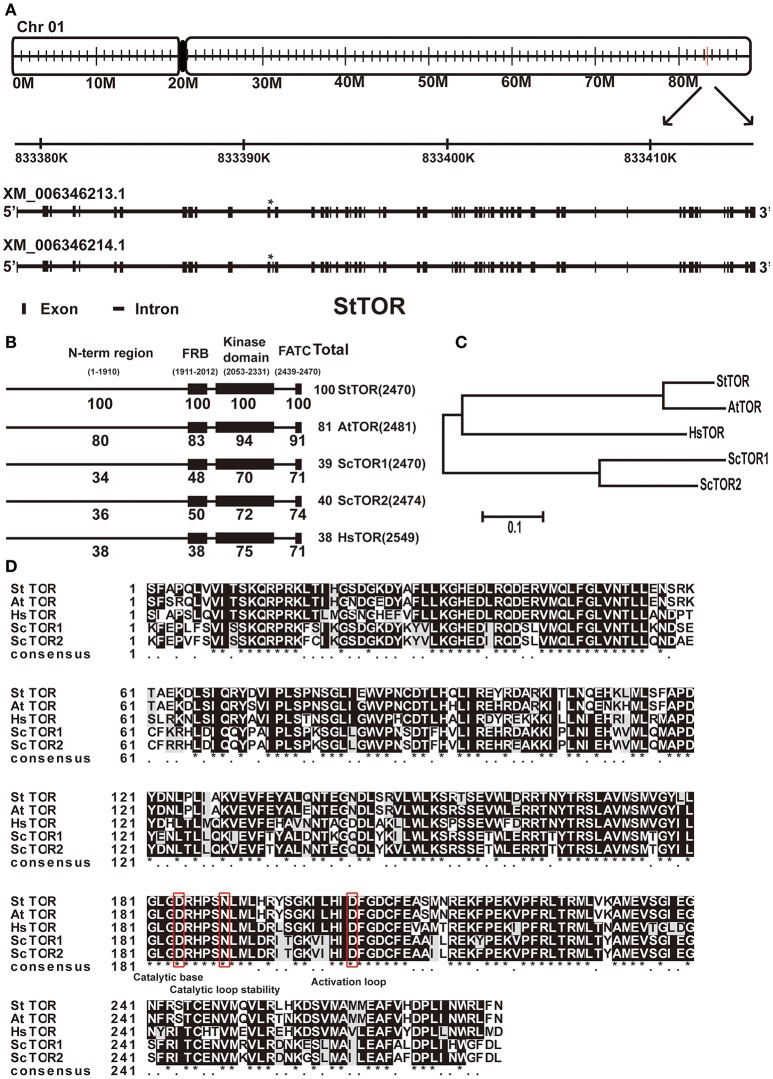
**Conservative evolution of StTOR in potato. (A)** The location of *StTOR* in chromosome and the difference of two different transcripts of *StTOR* in potato. The difference places of two transcripts were marked with ^*^. **(B,C)** Similarity comparison of protein and phylogenetic tree between StTOR and its homologs from other species (St, *Solanum tuberosum*, At, *Arabidopsis thaliana*, Sc, *Saccharomyces cerevisiae*, Hs, *Homo sapiens*). N-term region includes HEAT repeats domain and FAT domain. **(D)** Comparison of kinase domain of StTOR and its homologs from other species. The conserved sites (catalytic base, catalytic loop stability, and activation loop) were marked with red box.

The entire genome of a homozygous doubled-monoploid derived from a *Solanum tuberosum* group Phureja is sequenced and annotated (http://plants.ensembl.org/Solanum_tuberosum/Search/New?db=core; The Potato Genome Sequencing Consortium, 2011). The availability of the whole potato genome sequence allowed us to identify the putative components of TOR complex in potato. The full length of *Arabidopsis* TOR (AT1G50030.1) amino acid sequence was used to BLAST against the potato genome database. The unique putative *TOR* (*StTOR*) locus, locating on the Chromosome 1, was found in potato (Figure 1A). To confirm the sequence of *StTOR*, we amplified and cloned the full length CDS (coding DNA sequence) of *StTOR* from potato cultivar *Desiree*. The sequencing results showed that the CDS of *StTOR* is identical to the homologs of TOR in potato genome database, and the corresponding genome sequence of *StTOR* showed that it spans about 36 kb genomic region containing 55 exons and 54 introns and encodes a predicted ~280 KD protein with 2470 aa (XM_006346213.1; Figure 1A). All the five key domains of TOR were found in the StTOR protein with higher identities on main domains like the FRB and kinase domain (Figure 1B). Based on phylogenetic analysis, StTOR showed a closer evolutionary relationship with AtTOR than any other TOR proteins (Figure 1C). The catalytic base, catalytic loop stability and activation loop sites of TOR kinase domain are also conserved in potato, suggesting that the catalytic function of TOR in potato may be conservative, and TOR signaling may exist in potato (Figure 1D). Additionally, the other core components of TOR complex 1 (TORC1) such as RAPTOR and LST8 were also found in potato genome (Table 1). Furthermore, qPCR results showed that the expression of TOR complex was constitutive in potato seedlings (Figure S2D). However, RICTOR, a vital component of TORC2, was not found (Table 1), suggesting that the existence of a conserved TORC1 but not TORC2 in potato genome.

**Table 1:** **Screening of putative components of TOR complex in potato**.

***Hs***	***Sc***	***At***	***St***
**TORC1**			
mTOR	ScTOR1/2	AtTOR	StTOR-like (PGSC0003DMT400066098)
RAPTOR	Kog1	AtRaptor1A/1B	StRAPTOR-like (PGSC0003DMT400043895)
LST8	LST8	AtLST8-1/2	StLST8-like (LOC102590527)
**TORC2**			
mTOR	ScTOR2	AtTOR	StTOR-like
Rictor	Avo3	–	–
LST8	LST8	AtLST8-1/2	StLST8-like

*At, Arabidopsis thaliana; Hs, Homo sapiens; Sc, Saccharomyces cerevisiae; St, Solanum tuberosum*.

### ScFKBP12 can bridge the interaction between StTOR and rapamycin in potato

Classic “FKBP12-rapamycin-TOR” negative regulation system, an easy manipulated and universal accepted approach, has been successfully used for interpreting the function of TOR in yeast and mammals (Choi et al., 1996; Virgilio and Loewith, 2006; Benjamin et al., 2011). Choi et al. (1996) reported that rapamycin can interact with the FRB domain of TOR through close contacting with aromatic residues, such as Tyr^1934^, Phe^1935^, Trp^2001^, Tyr^2005^, Phe^2008^, Ser^1931^, Leu^1927^, Thr^1998^, and Asp^2002^ (sequence numbers followed by human TOR protein; Choi et al., 1996). We found these key AA residues were highly conserved between potato, *Arabidopsis* and human through aligning the amino acid sequence of FRB domain of TOR from different species (Figure S1A), indicating that the function of TOR in potato could be dissected by using the rapamycin-FKBP12-TOR negative regulation system.

Many detected plants, such as *Arabidopsis, Vicia faba*, Lotus (*Lotus japonicus*), Tobacco (*Nicotiana henthamiana*), Millet (*Panicum miliaceum*), and Rice (*Oryza sativa*) had no obvious growth defects after treating with rapamycin even at very high concentrations (up to 20 μM) in solid medium. In order to examine the functions of TOR signaling pathway in potato, rapamycin sensitivity assays were tested. Data showed that potato explants had no obvious growth defects after treating with rapamycin, even at very high concentrations (up to 20 μM) compared to DMSO (a dissolvent of rapamycin; Figures S1C,E,F).

**Figure 2:**
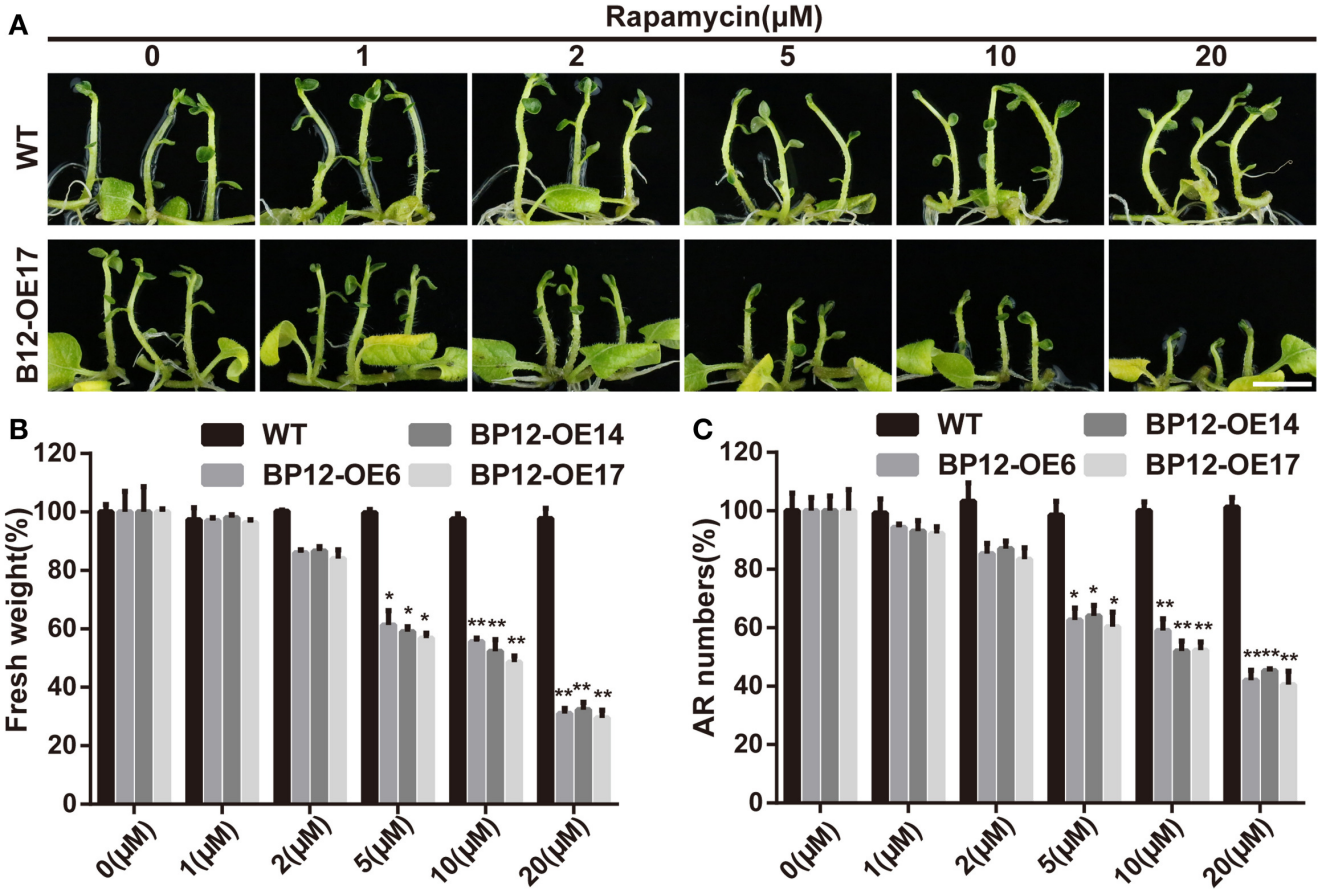
**ScFKBP12 can bridge the interaction of StTOR and rapamycin in potato. (A)** BP12-OE17 could respond to rapamycin but not WT, bar = 1 cm. **(B,C)** Rapamycin could effectively reduce the fresh weight and adventitious root (AR) numbers in BP12-OE lines. Asterisks denote Student's *t*-test significance compared with WT (^*^P < 0.05; ^**^P < 0.01). Each value represents the mean ± *SD* of 3 independent experiments.

Due to the highly conservation of TOR FRB domain, the resistance to rapamycin of potato might result from loss function of the StFKBP12, which bridge the interaction between rapamycin and TOR. The analysis of relative expression level of *StFKBP12* showed it was constitutive expression in potato, and the highest expression level was occurred in leaf, followed by shoot, and stem (Figure S1D). The constitutive expression of *StFKBP12* allows us to mine the changeable structure of StFKBP12 to interpret the resistance to rapamycin of potato. The alignment of FKBP12 AA sequences showed that the five important AA in forming the complex “rapamycin-FKBP12-TOR” in StFKBP12 were similar to *Arabidopsis*, which may affect its function (Figure S1B). Early studies showed that the FKBP12 protein from yeast and human could restore the rapamycin sensitivity in *Arabidopsis* (Mahfouz et al., 2006; Sormani et al., 2007; Ren et al., 2012). We therefore introduced yeast *FKBP12* (*ScFKBP12*), driving by a constitutive (cauliflower mosaic virus 35S) promoter, into potato. More than 20 individual transgenic lines (BP12-OE) were identified by leafy PCR (Figure S2A). No obvious morphological phenotypes appeared in any of transgenic plants. The *ScFKBP12* expression level was detected by qRT-PCR analysis (Figure S2B). Four plants with high *ScFKBP12* expression levels were examined by western blot (Figure S2C). Three of them were selected for rapamycin sensitivity assay. BP12-OE lines showed significant growth retardation on fresh weight and adventitious root formation (adventitious root numbers) compared with WT plants in a rapamycin dose dependent manner. With the increasing rapamycin concentration, the severer growth inhibition was observed (Figure 2).

### The growth of BP12-OE potato plants was significantly inhibited by combining asTORis with rapamycin

**Figure 3:**
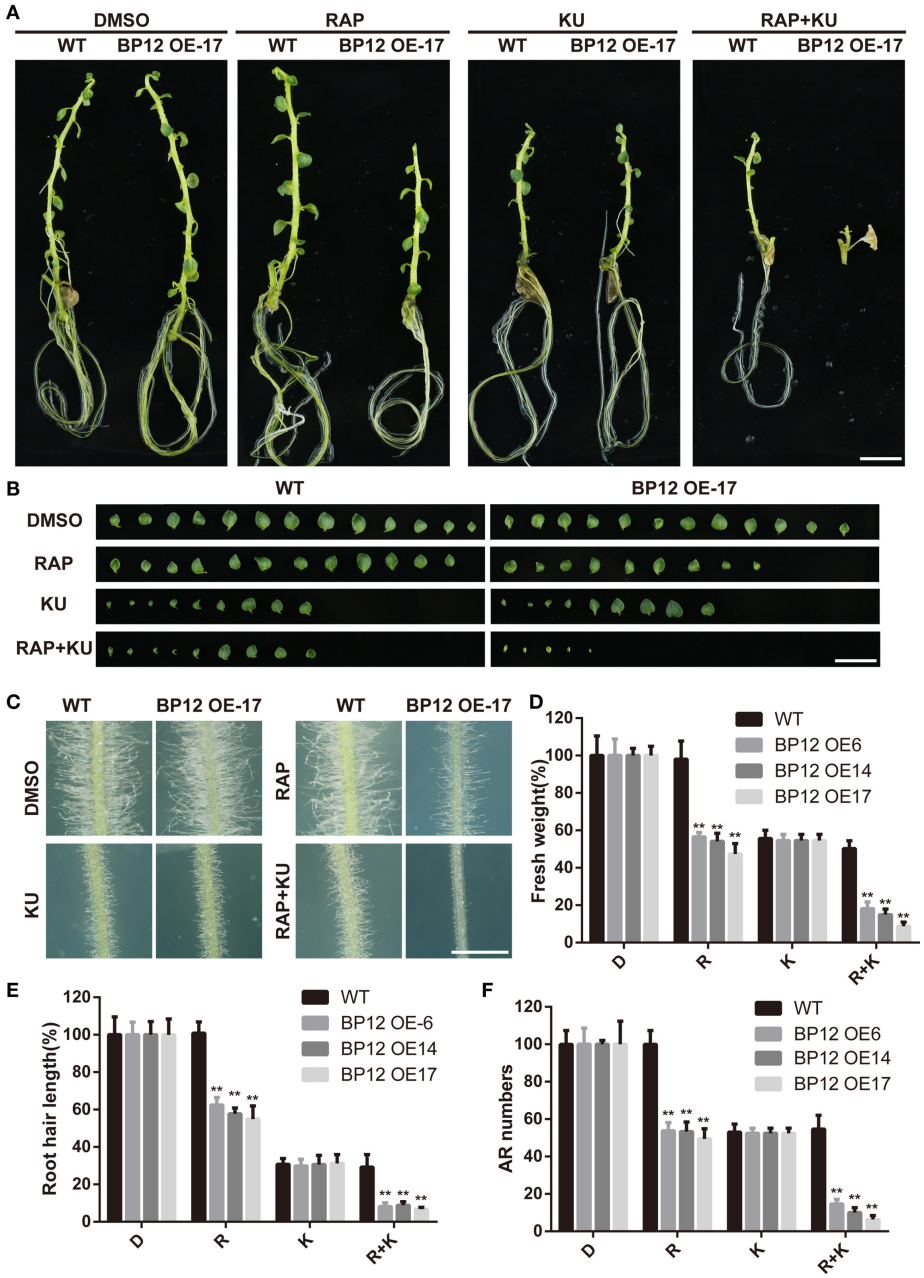
**The function of StTOR in regulation of potato seedlings growth. (A)** The phenotype of potato explants growth under medium with different TOR inhibitors or DMSO, bar = 1 cm. **(B)** The leaf growth of potato explants, bar = 1 cm. **(C,E)** The root hair growth and the root hair length of BP12-OE lines under different treatment. Asterisks denote Student's *t*-test significance compared with WT (^**^P < 0.01), bar = 1 mm. **(D,F)** The fresh weight and adventitious root (AR) numbers of BP12-OE lines. Asterisks denote Student's *t*-test significance compared with WT (^**^P < 0.01). Each value represents the mean ± *SD* of 3 independent experiments.

Our previous studies showed that the synergism inhibitory effects can be generated by combining the first and second generation inhibitors of TOR in *Arabidopsis* (Xiong et al., 2017). To better understand TOR signaling in potato, asTORis were employed to examine TOR signaling in potato. The dosage-dependent inhibitory effects of asTORis on the growth of cutting explants were observed and the IC50 of AZD and KU were about 2 and 10 μM in potato, respectively (Figure S3). Moreover, the combination of rapamycin and KU was examined in BP12-OE17 and WT and the fresh weight was measured on the 10th day. Through computing combination index (CI) values, data showed there were synergistic effects on inhibiting the potato growth by using of rapamycin + KU simultaneously (Figure S4). The results were similar to *Arabidopsis* BP12-2 (a well-established rapamycin sensitive transgenic material by introduced a ScFKBP12 gene into *Arabidopsis* in our previous study) treated with these drug combination (Ren et al., 2012; Xiong et al., 2017). And then the potato explants were subculture into MS medium with TOR inhibitors for 3 weeks to observed TOR functions in potato seedling growth. The results show that suppression of TOR could strongly inhibit the growth of potato seedlings (Figures 3A,D). Under rapamycin + KU treatment, the newly emerging leaves from BP12-OE17 potato seedling displayed a yellowed phenotype and the number of adventitious roots was also reduced (Figures 3A,B,F). Consistent with our previous studies in Arabidopsis, root hairs were also strongly suppressed under TOR inhibitors treatment in potato (Figures 3C,E; Ren et al., 2012; Deng et al., 2016).

### TOR plays important roles in adventitious root formation

**Figure 4:**
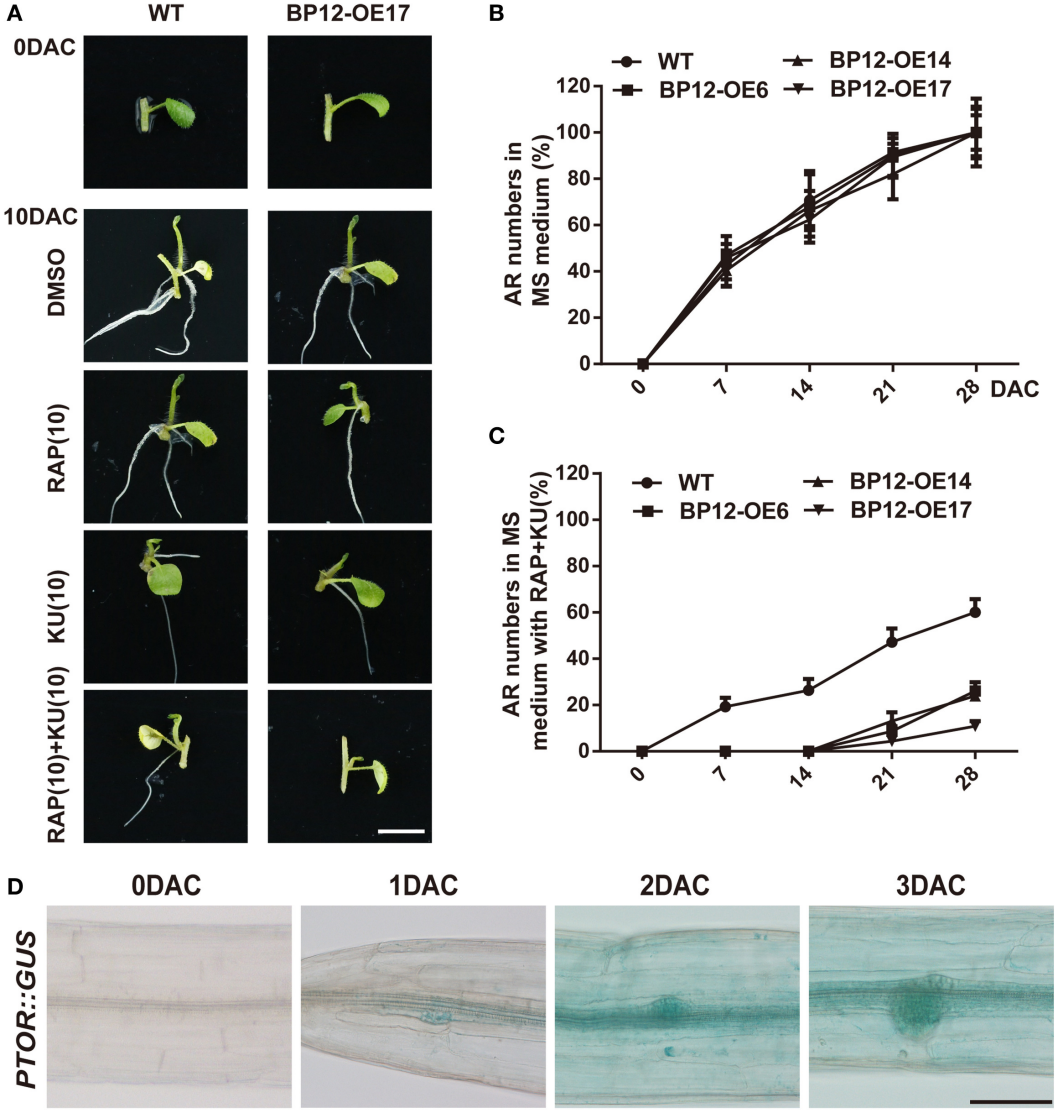
**StTOR functions in regulating adventitious root formation. (A)** Adventitious root formation of WT and BP12-OE17 explants under different TOR inhibitors in potato; DMSO was used as control, bar = 1 cm. **(B,C)** Adventitious root (AR) numbers in different time of WT and BP12-OE lines under DMSO and RAP + KU. **(D)** The *AtTOR* expression level during adventitious root formation in *Arabidopsis*, bar = 0.2 mm. Each value represents the mean ± *SD* of 3 independent experiments, for **(B,C)** *n* = 20, DAC means days after cutting.

To examine the functions of TOR in adventitious root formation, explants (stem cuttings) of potato seedlings were generated and grown on 1 × MS culture medium with different TOR inhibitors for 10 days. Data showed that adventitious root formation was retarded in BP12-OE cuttings compared with WT, when the cuttings grown on the medium with rapamycin (Figure 4A). Consistently, the synergistic inhibitory effect of rapamycin + KU was also observed in adventitious root formation of BP12-OE lines (Figure 4A). The adventitious roots hardly occurred in BP12-OE lines under rapamycin + KU treatment (Figures 4B,C). We further used the previously constructed *PTOR:: GUS* to observe the expression of *TOR* during adventitious root formation. The expression of *TOR* in the beginning of adventitious root formation has been induced, and then with the development of adventitious root primordium, the TOR expression increased gradually (Figure 4D). These results suggested TOR may play an important role in adventitious root formation.

### Transcriptome profiling of potato in response to TOR inhibitors

Next, the RNA-seq assay was performed to dissect the possible pathways associated with adventitious root formation which were regulated by TOR signaling in potato. The potato stem cuttings were treated with Rapamycin, KU and their combination RAP + KU for further RNA-seq assay. To investigate the early molecular events in response to TOR suppression in potato, the RNA were extracted from the samples treated with rapamycin, KU and their combination after 48 h, respectively, and then sequenced with Illumina Genome Analyzer (II). An overview of the sequencing and assembly was outlined in Table S3.

**Figure 5:**
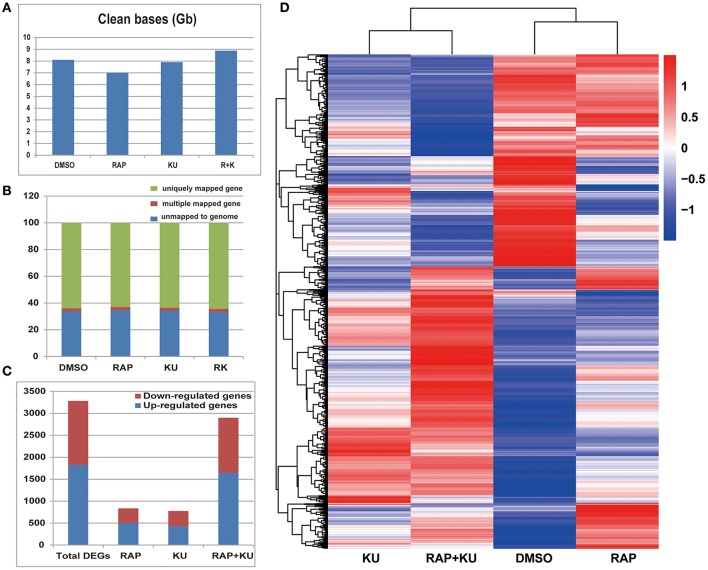
**The summary of basic information of the transcriptome data. (A)** The number of clean bases in the treatments DMSO, rapamycin, KU, and rapamycin + KU. **(B)** The proportion of total clean reads in the four transcriptome libraries that mapped to the reference diploid potato genome. **(C)** The DEGs of all three transcriptome data from rapamycin, KU, and rapamycin + KU compared with the control DMSO. **(D)** Hierarchical clustering of the DEGs in the treatments of rapamycin, KU, and rapamycin + KU and the control DMSO. The blue bands indicate low gene expression quantity; the red bands indicate high gene expression quantity.

After trimming and filtering, the number of clean bases in treatment of DMSO, rapamycin, KU and rapamycin + KU was 8.10, 7.01, 7.91, and 8.88 Gb, respectively (Figure 5A). We mapped the clean reads to the potato reference genome (The Potato Genome Sequencing Consortium, 2011). The proportion of total clean reads in the four transcriptome libraries mapped to the reference diploid potato genome ranged from 65.11 to 66.54% (Figure 5B). The abundance of all the genes (46,217) was normalized calculated by the expected number of fragments per kilobase of transcript sequence per millions base pairs sequenced (FPKMs) method using unique mapped reads (Table S4). Genes with FPKMs over 60 were considered to be expressed at a very high level, and genes with FPKMs in the interval 0–1 were considered to be present at very low levels or not to be expressed. The distributions of the expression levels of all the genes were similar for all four treatments. We found that about 46% of genes (46,217) were lowly expressed (FPKM ≥ 1), and more than 2,248 genes were highly expressed (FPKM > 60). To evaluate the validity of transcriptome sequencing analysis, six candidate genes were selected and detected by qRT-PCR. The expressions of six genes according to the qRT-PCR results agreed well with the data from Illumina sequencing analysis (Figure S8A).

The rapamycin + KU treatment group got the most DEGs (2,899), while the KU treatment group got the fewest (776) (Figure 5C). After getting rid of the genes with different expression tendency among rapamycin vs. DMSO, KU vs. DMSO, and rapamycin + KU vs. DMSO, the total of unique 3,281 DEGs were found (1,830 were up-regulated and 1,451 were down-regulated; Table S2). To observe the gene expression patterns, the hierarchical clustering of all the DEGs were performed based on the log_10_(FPKM + 1) of the four treatments (Figure 5D). GO and KEGG pathway analysis showed the conserved TOR functions in potato, such as participating cell wall restruction, photosynthesis, plant hormone signaling pathway etc., suggesting TOR is a core regulator of growth and development in potato (Tables S5, S6).

Further analysis revealed that a large number of genes associated with root development were affected in three treatment groups, particularly in the RAP + KU treatment group, including phytohormone, protein synthesis and degradation, cell division and cycle, cell wall restruction, and peroxidase (Tables S7–S10). The changes of auxin related genes are particularly evident in phytohormones related genes (Table S7), and the previous studies have showed there were some interconnections between auxin and TOR (Dinkova et al., 2000; Beltran-Pena et al., 2002; Schepetilnikov et al., 2013; Dong et al., 2015; Deng et al., 2016). We further examined potential crosstalk between TOR and auxin in the regulating of adventitious root formation.

### TOR could regulate adventitious root formation via TIR1/AFBs-mediated auxin signaling pathways in *arabidopsis*

The transcriptome data showed that the most DEGs were observed in auxin signaling pathway among all hormone signaling pathways. Table S7 showed that 39 genes associated with auxin signaling transduction were differentially expressed in RNA-seq data, implying that TOR played vital roles in auxin signaling pathway in potato (Table S7). *DR5::GUS* is a well-established and most commonly used auxin marker. In adventitious root formation, it has been found that auxin accumulation occurred in early stage (Liu et al., 2014; Rovere et al., 2016). In previous studies, we have generated DR5/BP12, a rapamycin sensitive *Arabidopsis* plant (Deng et al., 2016). To understand the relationship among auxin, TOR and adventitious root formation, DR5/BP12 seedling cuttings were treated with TOR inhibitors. Results showed auxin accumulation was significantly suppressed when TOR was inhibited in *Arabidopsis*. Especially when DR5/BP12 seedling cuttings were subjected to the medium with rapamycin + KU, adventitious root formation was completely inhibited and no GUS signal was detected (Figure S5A). In adventitious root formation, auxin accumulation mainly depends on the biosynthesis and polar transport of auxin which regulated by some important genes such as *YUCCA6, PIN1, ABCB19*, and *LAX3* (Sukumar et al., 2013; Della Rovere et al., 2015; Chen et al., 2016; Rovere et al., 2016). Therefore, the expression level of these genes under TOR inhibitor treatment were examined. The data showed that the expression of these genes were suppressed by TOR inhibitors, especially the combination of rapamycin, and KU (Figures S5B,C).

**Figure 6:**
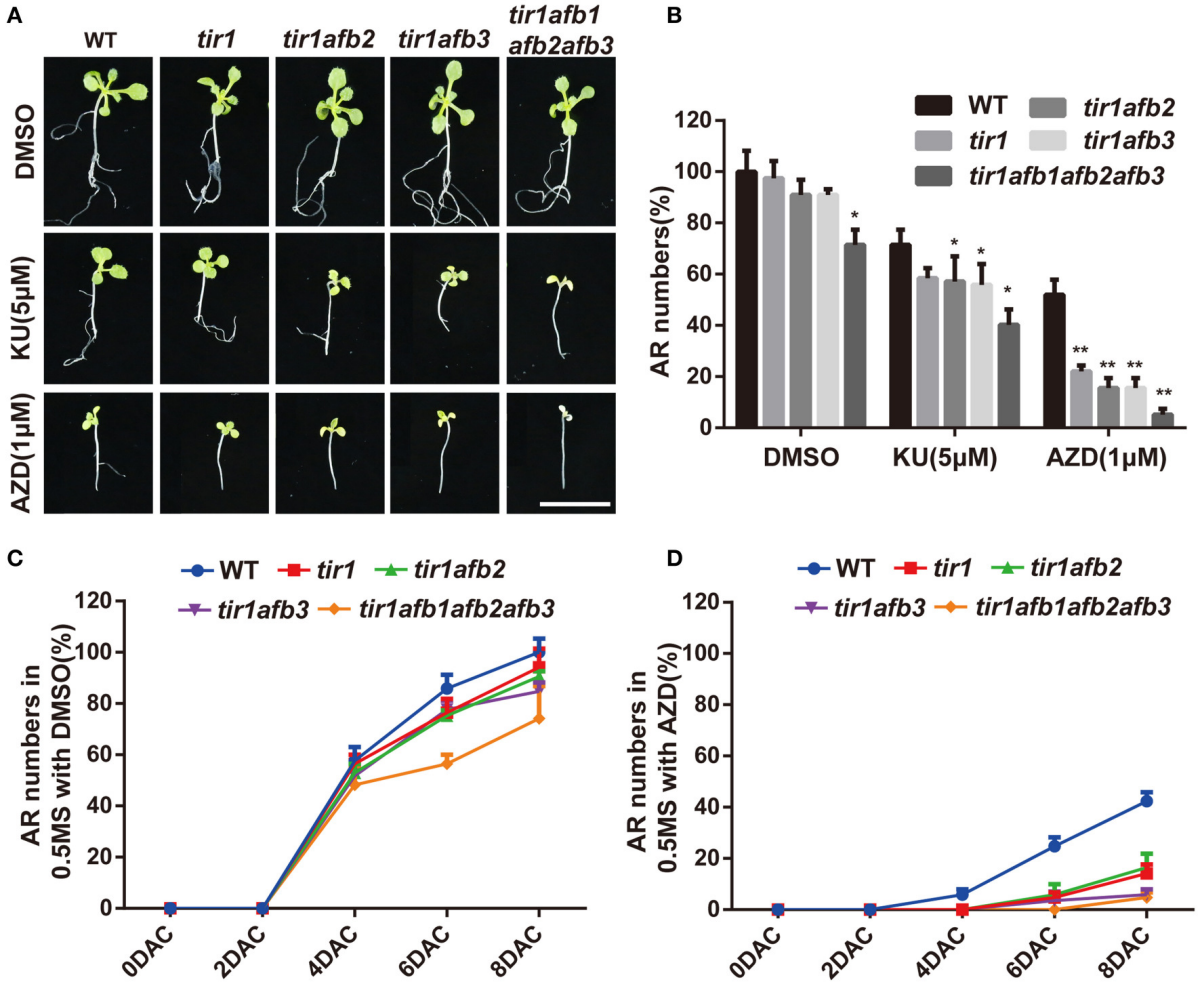
**The mutations of auxin receptors were sensitive to asTORis in ***Arabidopsis***. (A)** Adventitious root formation of WT and auxin receptor mutant under different asTORis treatment; DMSO was used as control, bar = 1 cm. **(B)** Adventitious root (AR) numbers of WT and auxin receptor mutants under different asTORis treatment; DMSO was used as control. **(C,D)** Adventitious root numbers in different time of WT and auxin receptor mutants under DMSO and AZD treatment. Asterisks denote Student's *t*-test significance compared with DMSO (^*^P < 0.05; ^**^P < 0.01). Each value represents the mean ± *SD* of 3 independent experiments, for **(C,D)** *n* = 30.

**Figure 7:**
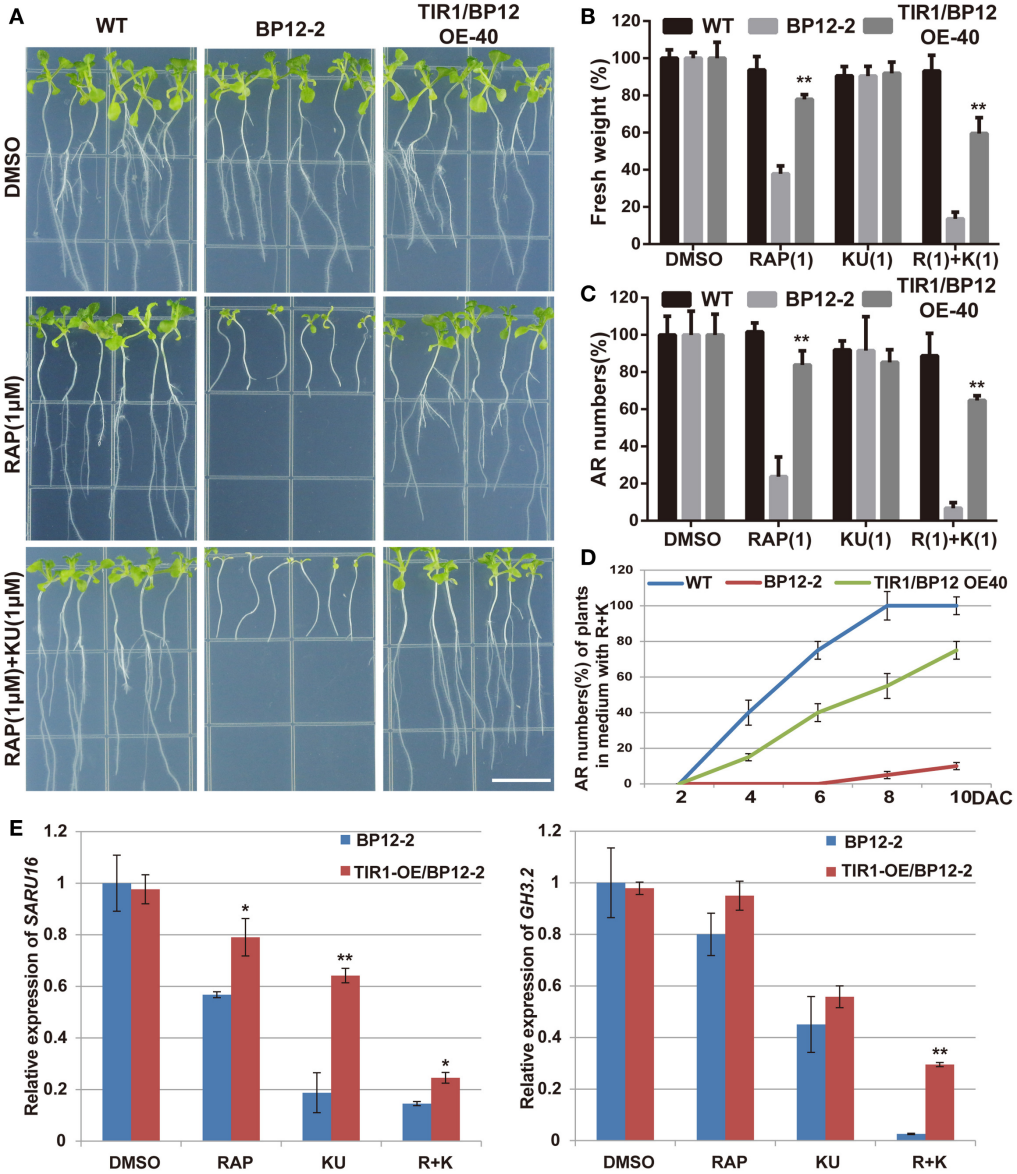
**The overexpression of ***AtTIR1*** can enhanced adventitious root formation ability of BP12-2 under TOR inhibitors treatment in ***Arabidopsis***. (A)** Adventitious root formation of WT, BP12-2, and TIR1/BP12 OE-40 under different TOR inhibitors treatment, bar = 1 cm. **(B,C)** The fresh weight and adventitious root (AR) numbers of WT, BP12-2, and TIR1 /BP12 OE-40 under different TOR inhibitors treatment, DMSO was used as control. **(D)** Adventitious root numbers in different time of WT and BP12-2 and TIR1/BP12 OE-40 under R + K treatment. **(E)** The expression of auxin primary response genes under TOR inhibitors treatment in BP12-2 and TIR1/BP12-OE40. Four days old BP12-2 and TIR1/BP12-OE40 seedlings grew in 0.5 MS medium with low light; then the root of seedlings was removed and stems were transferred to the medium containing TOR inhibitors [RAP (5μM), KU (5μM), RAP (5μM) + KU (5μM); DMSO was used as control] under normally growth condition 48 h. Asterisks denote Student's *t*-test significance compared with BP12-2 (^*^P < 0.05; ^**^P < 0.01). Each value represents the mean ± *SD* of three independent experiments, for **(D)** *n* = 30.

In previous studies, Transport Inhibitor Response 1 /Auxin Response F-box (TIR1/AFBs) were well-identified as auxin receptors through the biochemical and genetic approaches (Dharmasiri et al., 2005a,b; Kepinski and Leyser, 2005). In order to further understand the mechanism of TOR and auxin signal dependent adventitious root formation, we collected their single, double and quadruple mutants, *tir1, tir1afb2, tir1afb3*, and *tir1afb1afb2afb3*. The seeds of these mutants (*tir1, tir1afb2, tir1afb3*, and *tir1afb1afb2afb3*) and WT were grown on the 0.5 × MS in the dark to generate long hypocotyl for 4 days. Root-excised hypocotyls of mutants and WT were transferred to the mediums with AZD (1 μM), KU (5 μM) or DMSO under normal growth condition. Adventitious root formation was significantly inhibited when the mutants were growing on the medium with the asTORis (Figures 6A,B). Adventitious root formation was completely suppressed in quadruple *tir1afb1afb2afb3* plants grown on medium supplemented with TOR inhibitors (Figures 6C,D). Furthermore, we collected two gain-of-function mutant *axr2-1* and *axr3-1* which laid in the downstream of auxin receptors in auxin signaling pathways. AXR2 and AXR3 belong to AUX/IAA protein family and act as a repressor of auxin signaling transduction. These two mutant showed more sensitivity to TOR inhibitors compared to WT (Figures S7A,B). We therefore used AZD and KU to treat *HS:AXR3NT-GUS*, a well-established marker for detecting the degradation of AUX/IAA, to observe the effects of TOR inhibitors on auxin signaling transduction. When using the TOR inhibitors to treat *HS:AXR3-NT-GUS*, we observed that both the AXR3-GUS signal and GUS activities were stronger than the control (Figures S6B–D). These data indicated that the degradation of AUX/IAA was repressed and the auxin signaling transduction was inhibited when the activity of TOR was suppressed. Therefore, we proposed that TOR inhibitors may regulate the auxin signaling transduction by inhibiting the expression of *TIR1/AFBs* genes. And then, BP12-2 a well-established rapamycin sensitive line was treated with different TOR inhibitors to detect the expression level of *TIR1/AFBs* genes (Ren et al., 2012). The data show that *TIR1/AFBs* genes were down regulated by TOR inhibitors in BP12-2 (Figure S6A). To understand the genetic links between TOR and AtTIR1 (*Arabidopsis* TIR1), the *35S::TIR1* construct was introduced into BP12-2 to generate TIR1 overexpression lines (called TIR1/BP12 OE). More than 30 TIR1/BP12 OE lines were identified with leafy PCR, semi-qPCR, and western blot (Figures S7C,D). Three individual lines were selected to treat with TOR inhibitors. The data showed that the growth of TIR1/BP12-2 OE lines were significant better than BP12-2 under TOR inhibitors treatment (Figures S7E–G). Overexpression of *AtTIR1* in BP12-2 is able to rescue adventitious root formation of BP12-2 under TOR inhibitors treatment (Figures 7A–D). We also examined the expression of two auxin responsive genes, *GH3.2* and *SAUR16*. And data showed that overexpression of *AtTIR1* may increase the expression of these genes under TOR inhibitors treatment compared to BP12-2 (Figure 7E). These results indicate that overexpression of *AtTIR1* can enhance the auxin signaling transduction in BP12-2 under TOR inhibitors treatment to promote adventitious root formation.

**Figure 8:**
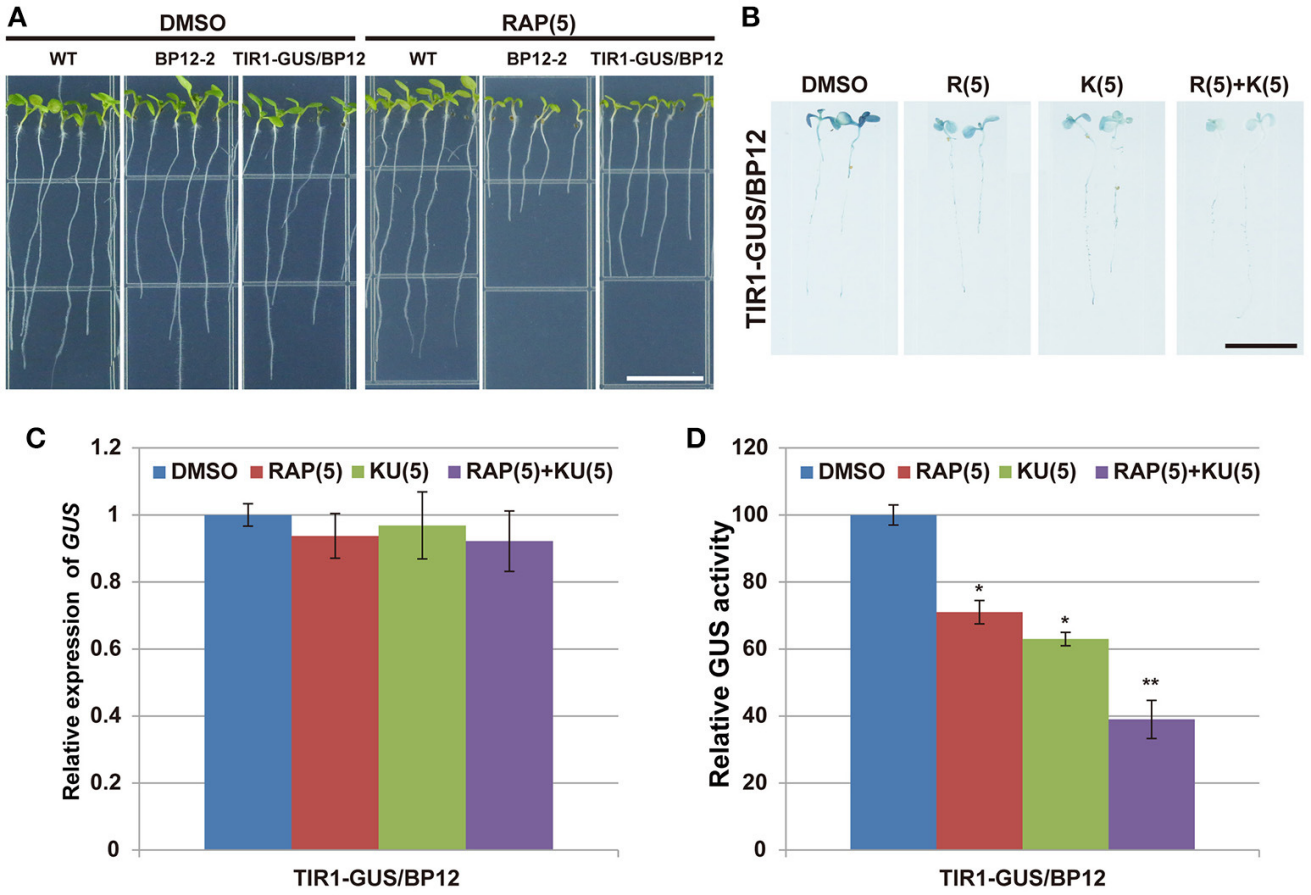
**TOR was involved in TIR1 stabilization in ***Arabidopsis***. (A)** The phenotype of TIR1-GUS /BP12 line in medium with rapamycin, bar = 1 cm. **(B)** The GUS staining of TIR1-GUS /BP12 line under different TOR inhibitors treatment; DMSO was used as control, bar = 1 cm. **(C,D)** The relative expression of GUS and the relative activity of GUS in TIR1-GUS /BP12 line under different TOR inhibitors treatment, DMSO was used as control. Asterisks denote Student's *t*-test significance compared with DMSO (^*^P < 0.05; ^**^P < 0.01). Each value represents the mean ± *SD* of three independent experiments.

Several recent studies have found that the regulation of TIR1/AFBs stability played an important role in auxin signal transduction (Yu et al., 2015; Wang et al., 2016). In order to further observe whether TOR plays a role in regulating the stability of TIR1/AFBs, P35S::TIR1-GUS was introduced into BP12-2 to generate TIR1-GUS overexpression lines (called TIR1-GUS/BP12). Consistently, TIR1-GUS/BP12 also grew better than BP12-2 under rapamycin treatment (Figure 8A). However, the GUS signals of TIR1-GUS/BP12 were significantly decreased under TOR inhibitors treatment (Figure 8B). We also tested the expression of *GUS* and GUS activity under different treatment. The data showed that the expression of GUS was not affected, but the activity of GUS was significantly reduced (Figures 8C,D), which may suggest that TIR1-GUS was degraded. These observations indicated that the stability of TIR was also regulated by TOR signaling.

### AtTIR1 rescues adventitious root formation in TOR signaling suppressed potato

**Figure 9:**
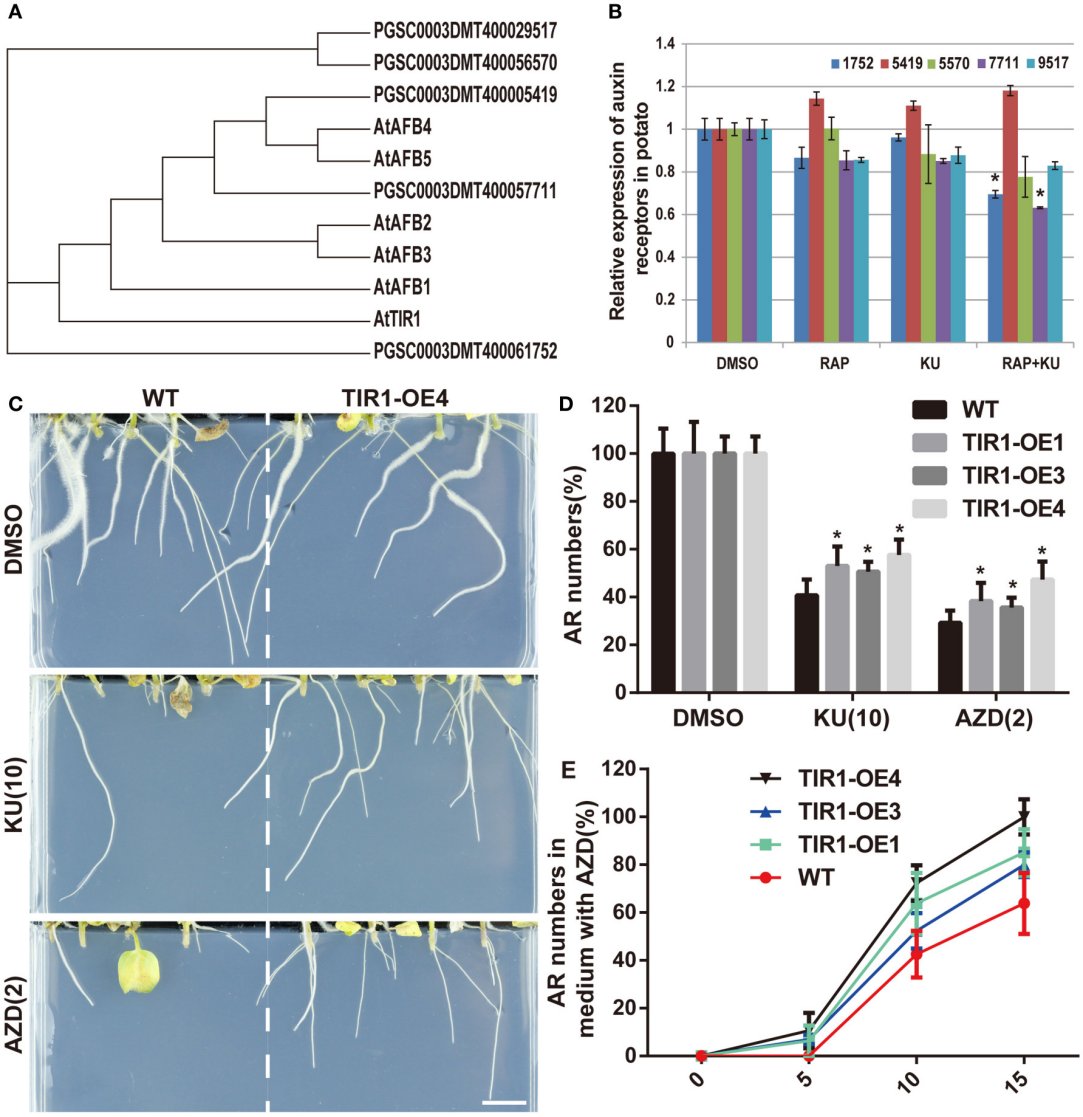
**The overexpression of ***AtTIR1*** in potato can enhance the adventitious root formation of potato explant under different asTORis treatment. (A)** Phylogenetic tree of auxin receptors in Arabidopsis and its homologs genes in potato. **(B)** The expression level of auxin receptor homologs genes in potato under TOR inhibitors treatment. Three weeks old BP12-OE17 seedlings grew in MS liquid medium and then were transfer to medium containing TOR inhibitors [RAP (10 μM), KU (10 μM), RAP (10 μM) + KU (10 μM); DMSO was used as control] under normally growth condition 48 h. **(C)** Adventitious root formation of WT and TIR1-OE4 under different asTORis treatment, bar = 1 cm. **(D)** Adventitious root (AR) numbers of WT and TIR1-OE4 under different asTORis treatment; DMSO was used as control. Asterisks denote Student's *t*-test significance compared with WT (^*^P < 0.05). **(E)** Adventitious root numbers in different time of WT and TIR1-OE lines under AZD treatment. Asterisks denote Student's *t*-test significance compared with DMSO (^*^P < 0.05). Each value represents the mean ± *SD* of three independent experiments, for **(E)** *n* = 20.

In order to investigate the relationship of TOR and auxin signaling pathway in potato adventitious root formation, we screened the homologous auxin receptors gene of potato by using AtTIR1 amino acid sequence in NCBI and potato genome database. Five candidates were found in potato genome and an evolutionary tree was constructed based on their amino acid sequences (Figure 9A). qPCR data showed that the expression level of these auxin receptor-like genes were also affected by TOR inhibitors (Figure 9B). To examine whether AtTIR1 can overcomes the inhibitory effect of asTORis on adventitious root formation in potato, *AtTIR1* was introduced into potato to generate AtTIR1-OE lines. More than 10 individual lines was generated and identified with leafy PCR and semi-qPCR (Figures S8B,C). Three individual lines were treated with asTORis to observe their adventitious root formation ability. The results showed that AtTIR1-OE lines were partially overcomes the inhibitory effect of asTORis and able to promote adventitious root formation under asTORis treatment compared with WT (Figures 9C–E). Taken together, these results showed that TOR and auxin had a closely relationship in regulating adventitious root formation, and its mechanism may be conserved evolution in potato and *Arabidopsis*.

## Discussion

The plant TOR research could trace back to 1998, when Xu et al. reported the *V. faba* FKBP12 could not mediate the action of FK506 and rapamycin to combine with TOR (Xu et al., 1998). Four years later (2002), Menand et al. firstly identified a single *TOR* gene encoding a protein able to complex with yeast FKBP12 in *Arabidopsis*; however, the *Arabidopsis* vegetative growth is insensitive to rapamycin (Menand et al., 2002). Most planta species including *Arabidopsis, Oryza sativa, Nicotiana tabacum*, and *Brassica napus* etc. were resistant to rapamycin (Xu et al., 1998; Montane and Menand, 2013). In this study, a single TOR was identified in potato through scanning the whole potato genome and sequencing of StTOR derived from *Desiree*. Similar to most examined planta species, potato seedlings showed insensitivity to rapamycin even the concentration up to 20 μM. The rapamycin-sensitive potato BP12-OE lines were generated by overexpression of yeast *FKBP12*. While rapamycin only partially inhibited TOR activity and displayed a plateau effect (Sormani et al., 2007; Ren et al., 2012; Deng et al., 2016; Xiong et al., 2017). AsTORis could inhibit both TORC1 and TORC2 in a dose-dependent manner in cancer therapy (Benjamin et al., 2011). Recent studies showed that asTORis can efficiently inhibit plants growth via blocking TOR activities (Montane and Menand, 2013; Xiong et al., 2013, 2017; Deng et al., 2016). Xiong et al. found that the combination of rapamycin and asTORis showed synergistic inhibitory effects on TOR activities in BP12-2 (Xiong et al., 2017). Here, the synergistic inhibition effects of rapamycin and KU were also observed in potato BP12-OE lines. Adventitious root formation was strongly retarded in potato and *Arabidopsis* under TOR inhibitors treatment, and the expression of TOR increased gradually with the development of adventitious roots, suggesting TOR was involved in adventitious root formation in potato.

The transcriptome data affirmed that TOR play vital roles in photosynthesis, phytohormone signaling pathway, ribosomal biogenesis, cell wall restruction, autophagy, ubiquitin, etc. in potato, which is similar to the analysis of previous expression profiling under TOR inhibition or activation. Further data mining of transcriptome revealed that a large number of DEGs was involved in critical pathways, such as cell cycle, cell wall restruction, protein synthesis, and degradation, peroxidase activity, plant hormone signaling transduction and especially the auxin signaling pathways, participated in adventitious root formation. That is similar to the previous transcriptome profiling about adventitious root or lateral root formation at different crucial stages (Brinker et al., 2004; Himanen and Beeckman, 2004; Péret et al., 2009; Majer et al., 2012; Du et al., 2016). For example, the peroxidase has been reported to be close related to the formation of adventitious root (Wang et al., 2015). Peroxidase activity (GO: 0004601) with 39 genes was dominant in the GO MF based on the 1,122 down-regulated DEGs (Table S8). Also, the DEGs related to protein synthesis and degradation were detected, such as 29 translation related genes (25 down-regulated and 4 up-regulated genes), 30 ubiquitin related genes (7 down-regulated and 23 up-regulated genes), and 5 up-regulated autophagy genes (Table S9). Ubiquitin can be attached to proteins and labeled them for final degradation. Autophagy is a process for the turnover and recycling of intracellular large macromolecules. Protein synthesis and degradation play vital roles in tissue regeneration and organogenesis. In addition, at the cellular level, lateral root formation is divided into two major steps, the degradation of overlaying cells and the reorganization of new cells (Péret et al., 2009). In this process, cell cycle, cell division, cell wall restruction, and biogenesis are very crucial steps. Total of 41 and 14 DEGs were detected in the cell cycle (GO: 0007049) and cell division (GO: 00051301), respectively (Table S10A-B). More than 100 DEGs were involved in cell wall restruction and biogenesis, which encoded expansion, extensin, xyloglucan endotransglucosylase/hydrolase protein, pectin lyase, cellulose synthase etc. in the transcriptome data (Table S10C). Importantly, it has been reported that phytohormones, particular auxin, are crucial adventitious root formation (Bellini et al., 2014). In our data, plant hormone signaling transduction KEGG pathway is one of the most significantly enriched pathways. A total of 75 DEGs were identified to be involved in six phytohormone signaling transduction pathways (Table S7). The number of DEGs in the auxin signaling transduction pathway was the highest (39 DEGs), including auxin responsive proteins (27), transport carrier (5), and regulation protein (7). The auxin responsive proteins GH3 (Gretchen Hagen 3) and SAUR (Small auxin up RNAs) were associated with cell enlargement and plant growth (Chen et al., 2014; Feng et al., 2015).

The relationship between auxin and TOR has been reported by different groups previously (Dinkova et al., 2000; Beltran-Pena et al., 2002; Schepetilnikov et al., 2013; Dong et al., 2015; Deng et al., 2016). TOR was response to auxin to regulate translation reinitiation (Schepetilnikov et al., 2013). Many phytohormone (auxin, ABA, Ethylene, etc.) signaling associated genes are differentially expressed in *Arabidopsis* seedlings treated with AZD (one of asTORis) (Dong et al., 2015). The TOR inhibitors could interfere with auxin redistribution in root tips and root gravitropic responses (Deng et al., 2016). In this study, after TOR inhibitors treatment, a huge number of genes involved in phytohormone signaling pathway were differentially expressed (Table S7), and auxin related genes were most differentially expressed. To further confirm the triangle relationship among TOR, auxin and adventitious root formation, BP12-2, DR5/BP12, auxin receptor mutants (*tir1, tir1afb2, tir1afb3, and tir1afb1afb2afb3*) and TIR1/BP12-2-OE lines were generated or used in the present study. Both the number of adventitious root and the accumulation of auxin in the adventitious root primordia were significantly reduced when the TOR was inactive in the auxin receptor mutants and DR5/BP12 compared to control, respectively. Overexpression of *AtTIR1* could partially rescue adventitious root formation defects resulting from TOR inhibitors in *Arabidopsis* and potato. All the results suggested that there were tight links between TOR and auxin signaling in adventitious root formation in *Arabidopsis* and potato (Figure S9). *YUCCA6, ABCB19, PIN1*, and *LAX3* which has important functions in auxin synthesis and accumulation were strongly affected by TOR signaling. During the adventitious root primordium formation, TOR could regulate the expression of auxin receptors and the stability of TIR1 protein to modulate auxin signaling dependent cell differentiation or proliferation. TOR may integrate multiple environmental and endogenous factors to regulate adventitious root formation through crosstalk with auxin signaling.

## Author contributions

MR, KD, and WW designed the experiments. KD, WW, KW, LF, FX, and SF performed the experiments. MR, PD, SZ, BW, and JZ analyzed the data. MR, KD, and PD wrote the manuscript text.

### Conflict of interest statement

The authors declare that the research was conducted in the absence of any commercial or financial relationships that could be construed as a potential conflict of interest.

## Supplementary material

Figure S1 **Conservative evolution of StTOR FRB domain and StFKBP12 in potato. (A)** Comparison of FRB domain of StTOR and homologs from other organisms. **(B)** Comparison of StFKBP12 and homologs from other organisms. **(C)** The rapamycin sensitivity test of potato explants, bar = 1 cm. **(D)** The relative expression level of *StFKBP12* in different tissues of potato. **(E,F)** The fresh weight and adventitious root (AR) numbers of WT under rapamycin treatment. Each value represents the mean ± *SD* of three independent experiments.

Figure S2 **The identification of transgenic BP12-OE lines in potato and relative expression of TOR complex in different tissue of potato. (A)** The leaf PCR identification of transgenic BP12-OE lines in potato. **(B)** qRT-PCR to detect relative expression level of *ScFKBP12* in BP12-OE lines in potato. **(C)** Western blot to detect relative content of ScFKBP12 in BP12-OE lines in potato. **(D)** The relative expression level of StTOR complex 1 in different tissues of potato. Each value represents the mean ± *SD* of three independent experiments.

Figure S3 **AsTORis inhibit seedling growth and adventitious root formation of potato. (A)** The growth of BP12-OE17 line could be inhibited by asTORis KU and AZD in a dose dependent manner, bar = 1 cm. **(B,C)** AsTORis can effectively reduce the fresh weight and adventitious root (AR) numbers in BP12-OE lines. Asterisks denote Student's *t*-test significance compared with WT (^*^*P* < 0.05; ^**^*P* < 0.01). Each value represents the mean ± *SD* of three independent experiments.

Figure S4 **Rapamycin and asTORis synergistically inhibit seedling growth of potato. (A)** The inhibition effect of rapamycin, KU and their combined treatment of BP12-OE17 line, bar = 1 cm. **(B)** Rapamycin and asTORis could synergistically reduce the fresh weight in BP12-OE line. **(C)** Fa-CI curve shows synergism (CI < 1) between rapamycin and KU. **(D)** Rapamycin and asTORis showed no synergistically effects in the regulating of the fresh weight in WT, bar = 1 cm. Asterisks denote Student's *t*-test significance compared with singly used rapamycin or KU (^*^*P* < 0.05; ^**^*P* < 0.01). Each value represents the mean ± *SD* of three independent experiments. Combination index (CI) was defined by Chou and CI values were calculated using the CompuSyn software program (ComboSyn Inc., Paramus, NJ, USA), CI > 1 represents antagonism, CI = 1 indicates additive effects, and CI < 1 indicates synergism (Chou and Talalay, 1984; Chou, 2006).

Figure S5 **TOR was involved in auxin synthesis and polar transport during adventitious root formation in ***Arabidopsis***. (A)** DR5 signal in adventitious root formation was strongly inhibited by TOR inhibitors, bar = 0.2 mm. **(B**,**C)** the expression of auxin synthesis and polar transport related genes under TOR inhibitors treatment. Four days old BP12-2 seedlings grew in 0.5 MS medium with low light; then the root of seedlings were removed and stems were transferred to the medium with TOR inhibitors [RAP (5 μM), KU (5 μM), RAP (5 μM) + KU (5 μM); DMSO was used as control] under normally growth condition for 48 h. Asterisks denote Student's *t*-test significance compared with DMSO (^*^*P* < 0.05; ^**^*P* < 0.01). Each value represents the mean ± *SD* of three independent experiments.

Figure S6 **The expression of ***TIR1/AFBs*** and the degradation of AXR3NT-GUS under different TOR inhibitors treatment in ***Arabidopsis***. (A)** The expression of *TIR1/AFBs* during different TOR inhibitors treatment. Four days old BP12-2 seedlings grew in 0.5 MS medium with low light; then the root of seedlings were removed and stems were transferred to the medium containing TOR inhibitors [RAP (5 μM), KU (5 μM), RAP (5 μM) + KU (5 μM); DMSO was used as control] under normally growth condition for 48 h. **(B)** The GUS staining of *HS:: AXR3NT-GUS* under different TOR inhibitors treatment, KU(10 μM), AZD(2 μM), and DMSO was used as control, bar = 1 mm. **(C,D)** The relative expression of GUS and the relative activity of GUS in *HS:: AXR3NT-GUS* under different TOR inhibitors treatment; DMSO was used as control. Asterisks denote Student's *t*-test significance compared with control (^*^*P* < 0.05; ^**^*P* < 0.01). Each value represents the mean ± *SD* of three independent experiments.

Figure S7 **The auxin signal transduction was affect by TOR inhibitors in ***Arabidopsis***. (A,B)** The gain-of-function mutants *axr2-1* and *axr3-1* were sensitivity to asTORis compared to WT, bar = 1 cm. **(C,D)** The leaf PCR, semi-qPCR and western blot identification of TIR1/BP12-OE lines. **(E)** The phenotype of TIR1 /BP12 OE lines in medium with different TOR inhibitors, bar = 1 cm. **(F,G)** The fresh weight and lateral root numbers of WT, BP12-2 and TIR1-OE/BP12 lines under different TOR inhibitors treatment; DMSO was used as control. Asterisks denote Student's *t*-test significance compared with BP12-2 (^*^*P* < 0.05; ^**^*P* < 0.01). Each value represents the mean ± *SD* of three independent experiments.

Figure S8 **The validation of RNA-Seq data by qRT-PCR, and identification of potato TIR1-OE lines. (A)** The validation of RNA-Seq data by qRT-PCR, and gene ID numbers are listed in Table S1. **(B,C)** The leaf PCR and semi-qPCR identification of potato transgenic lines of TIR1-OE.

Figure S9 **A model for the crosstalk between TOR signaling and auxin signaling during adventitious root formation**.

Table S1 **Primers were used in this study and related gene ID in this study**.

Table S2 **The differentially expressed genes in the BP12-OE17 treated with TOR inhibitors**.

Table S3 **Summary of the sequence assembly after Illumina sequencing**.

Table S4 **Distribution of gene expression in the BP12-OE17 under the four different treatments**.

Table S5 **GO enrichment analysis based on the 3281 differentially expressed genes in potato BP12-OE17 treated with different TOR inhibitors and DMSO**.

Table S6 **KEGG pathway enrichment analysis based on the 3281 differentially expressed genes in potato BP12-OE17 treated with different TOR inhibitors and DMSO**.

Table S7 **Differentially expressed genes related to phytohormone signal pathway in potato under the treatment of rapamycin, KU, and rapamycin + KU**.

Table S8 **Differentially expressed genes related to peroxidase in potato under the treatment of rapamycin, KU, and rapamycin + KU**.

Table S9 **DEGs involved in the protein synthesis and degradation in potato under the treatment of TOR inhibitors**.

Table S10 **DEGs participated in cell wall restruction**.
