# Supplementary figures and images for: The TOR Pathway Is Involved in Adventitious Root Formation in Arabidopsis and Potato
Source: Front Plant Sci. 2017 May 12;8:784. doi: 10.3389/fpls.2017.00784 (PMC5427086; doi:10.3389/fpls.2017.00784)

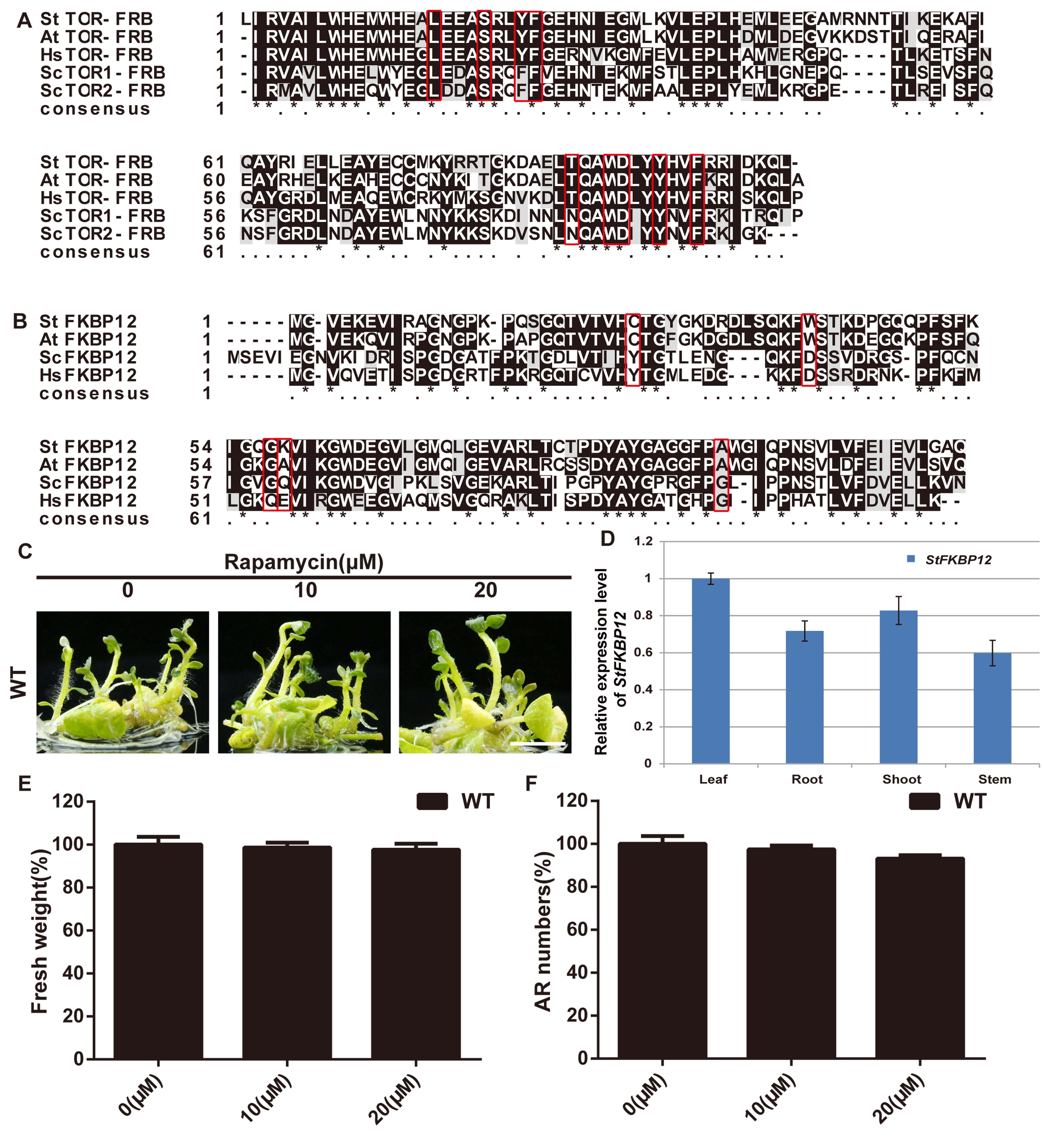

Supplement: Figure S1 — Conservative evolution of StTOR FRB domain and StFKBP12 in potato. (A) Comparison of FRB domain of StTOR and homologs from other organisms. (B) Comparison of StFKBP12 and homologs from other organisms. (C) The rapamycin sensitivity test of potato explants, bar = 1 cm. (D) The relative expression level of StFKBP12 in different tissues of potato. (E,F) The fresh weight and adventitious root (AR) numbers of WT under rapamycin treatment. Each value represents the mean ± SD of three independent experiments. [file Image1.JPEG]

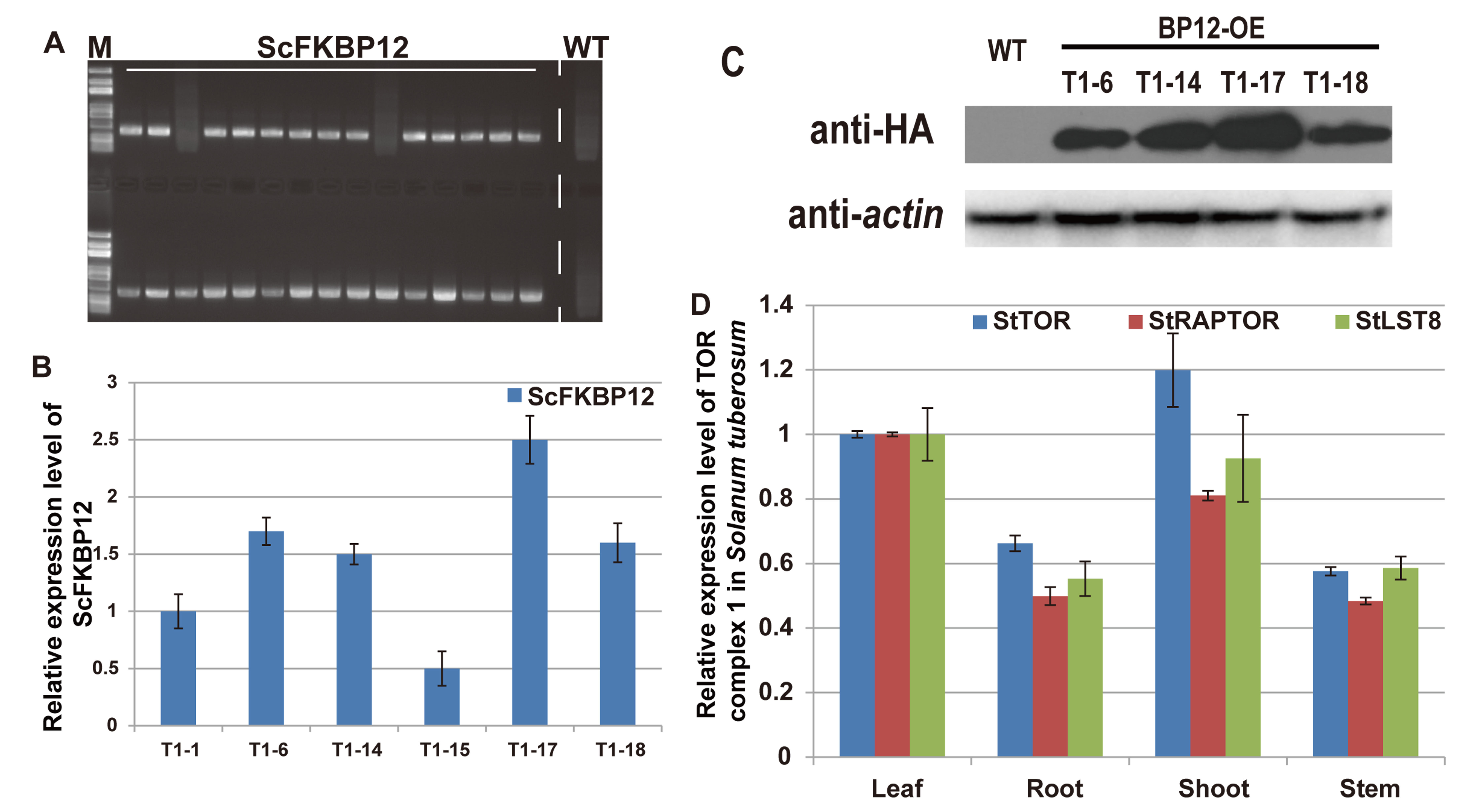

Supplement: Figure S2 — The identification of transgenic BP12-OE lines in potato and relative expression of TOR complex in different tissue of potato. (A) The leaf PCR identification of transgenic BP12-OE lines in potato. (B) qRT-PCR to detect relative expression level of ScFKBP12 in BP12-OE lines in potato. (C) Western blot to detect relative content of ScFKBP12 in BP12-OE lines in potato. (D) The relative expression level of StTOR complex 1 in different tissues of potato. Each value represents the mean ± SD of three independent experiments. [file Image2.JPEG]

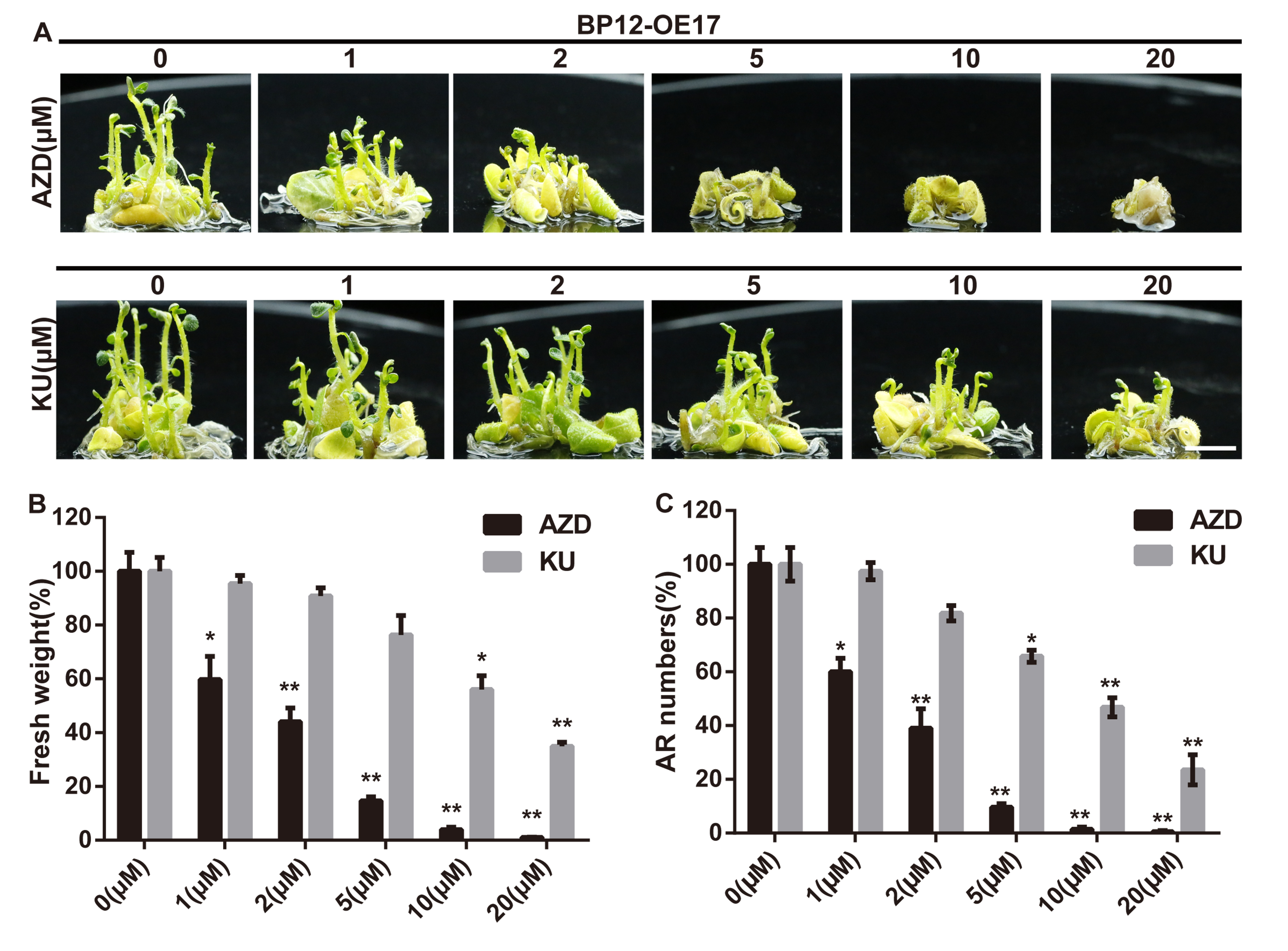

Supplement: Figure S3 — AsTORis inhibit seedling growth and adventitious root formation of potato. (A) The growth of BP12-OE17 line could be inhibited by asTORis KU and AZD in a dose dependent manner, bar = 1 cm. (B,C) AsTORis can effectively reduce the fresh weight and adventitious root (AR) numbers in BP12-OE lines. Asterisks denote Student's t-test significance compared with WT (*P < 0.05; **P < 0.01). Each value represents the mean ± SD of three independent experiments. [file Image3.JPEG]

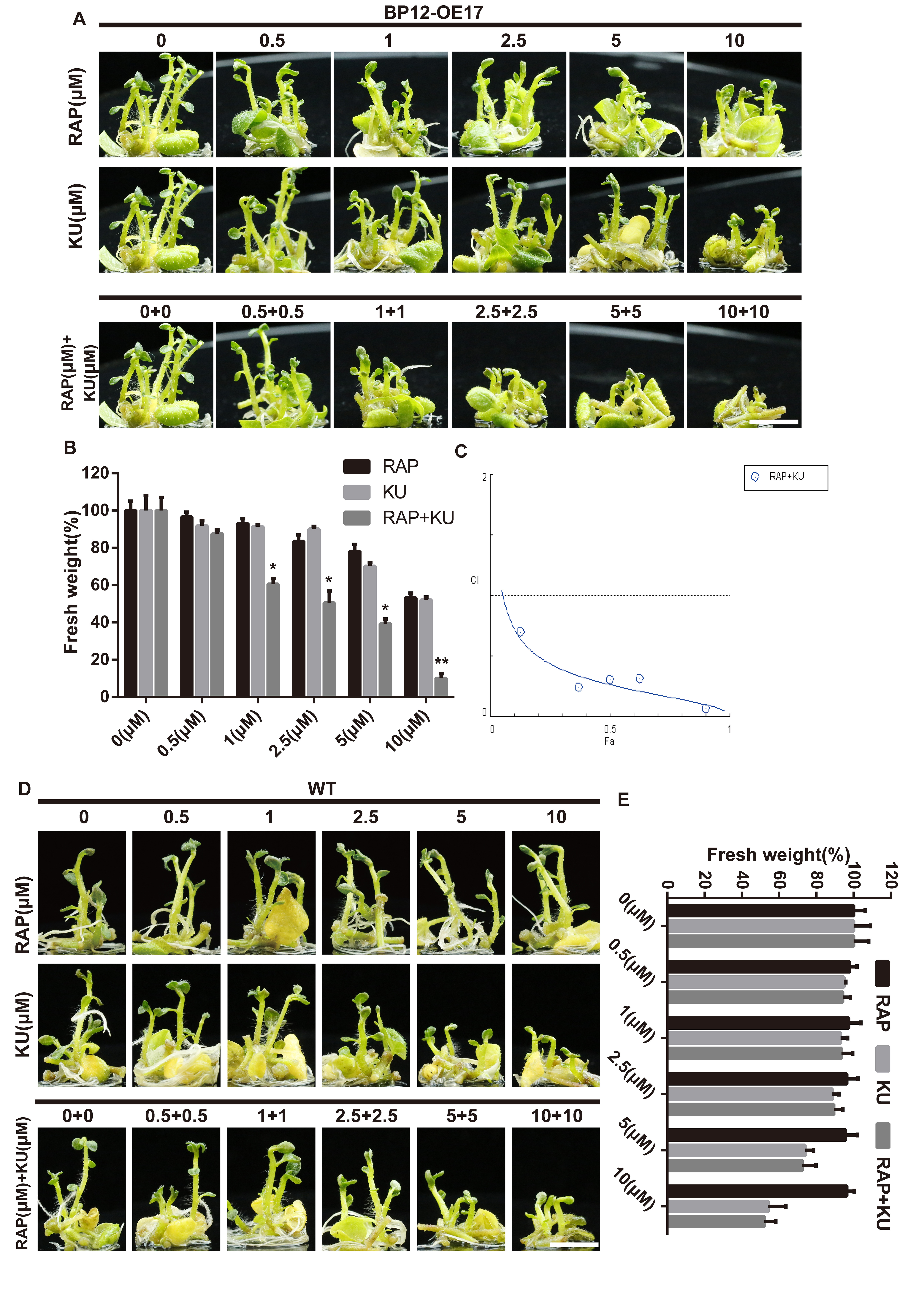

Supplement: Figure S4 — Rapamycin and asTORis synergistically inhibit seedling growth of potato. (A) The inhibition effect of rapamycin, KU and their combined treatment of BP12-OE17 line, bar = 1 cm. (B) Rapamycin and asTORis could synergistically reduce the fresh weight in BP12-OE line. (C) Fa-CI curve shows synergism (CI < 1) between rapamycin and KU. (D) Rapamycin and asTORis showed no synergistically effects in the regulating of the fresh weight in WT, bar = 1 cm. Asterisks denote Student's t-test significance compared with singly used rapamycin or KU (*P < 0.05; **P < 0.01). Each value represents the mean ± SD of three independent experiments. Combination index (CI) was defined by Chou and CI values were calculated using the CompuSyn software program (ComboSyn Inc., Paramus, NJ, USA), CI > 1 represents antagonism, CI = 1 indicates additive effects, and CI < 1 indicates synergism (Chou and Talalay, 1984; Chou, 2006). [file Image4.JPEG]

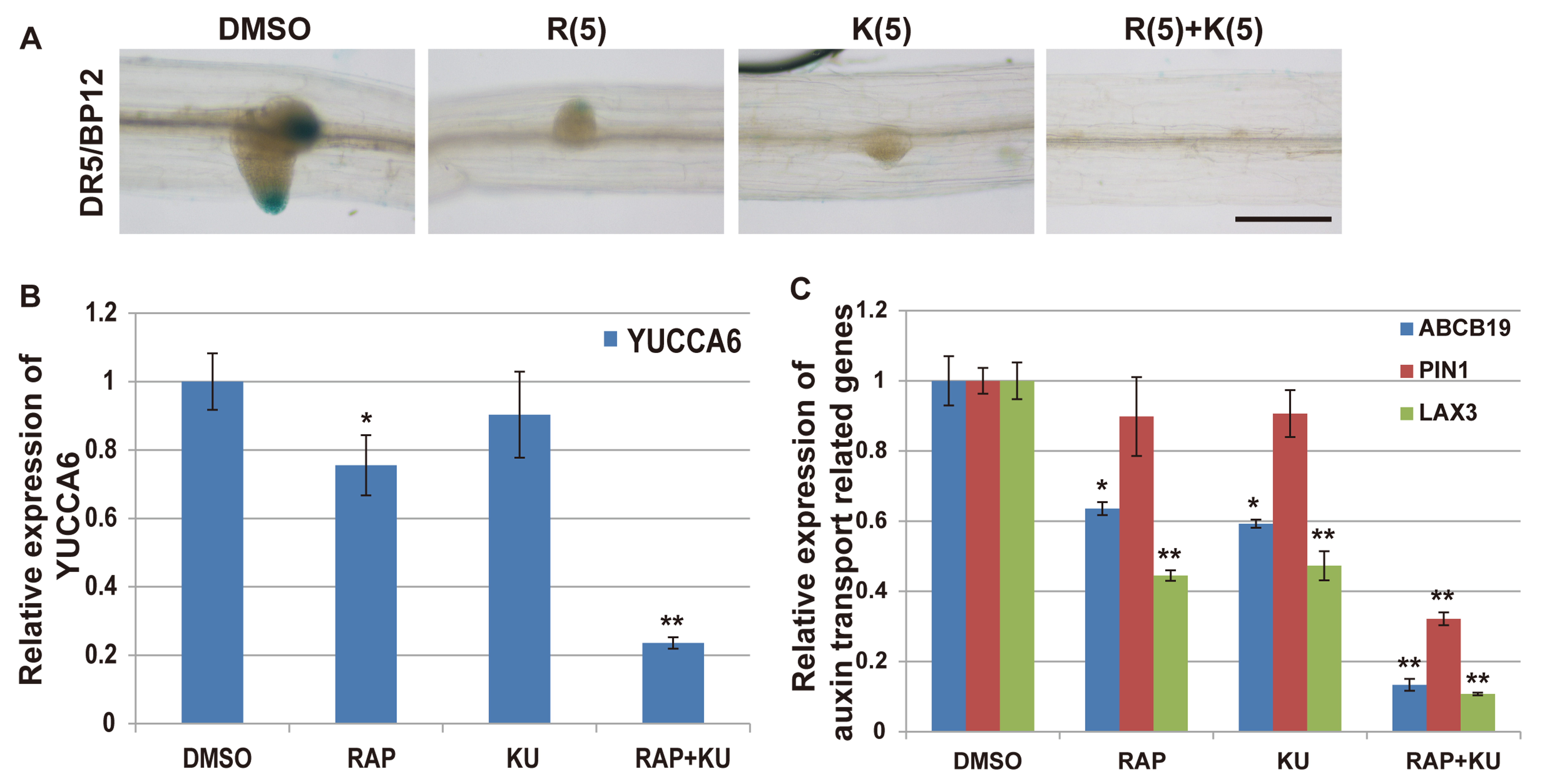

Supplement: Figure S5 — TOR was involved in auxin synthesis and polar transport during adventitious root formation in Arabidopsis. (A) DR5 signal in adventitious root formation was strongly inhibited by TOR inhibitors, bar = 0.2 mm. (B,C) the expression of auxin synthesis and polar transport related genes under TOR inhibitors treatment. Four days old BP12-2 seedlings grew in 0.5 MS medium with low light; then the root of seedlings were removed and stems were transferred to the medium with TOR inhibitors [RAP (5 μM), KU (5 μM), RAP (5 μM) + KU (5 μM); DMSO was used as control] under normally growth condition for 48 h. Asterisks denote Student's t-test significance compared with DMSO (*P < 0.05; **P < 0.01). Each value represents the mean ± SD of three independent experiments. [file Image5.JPEG]

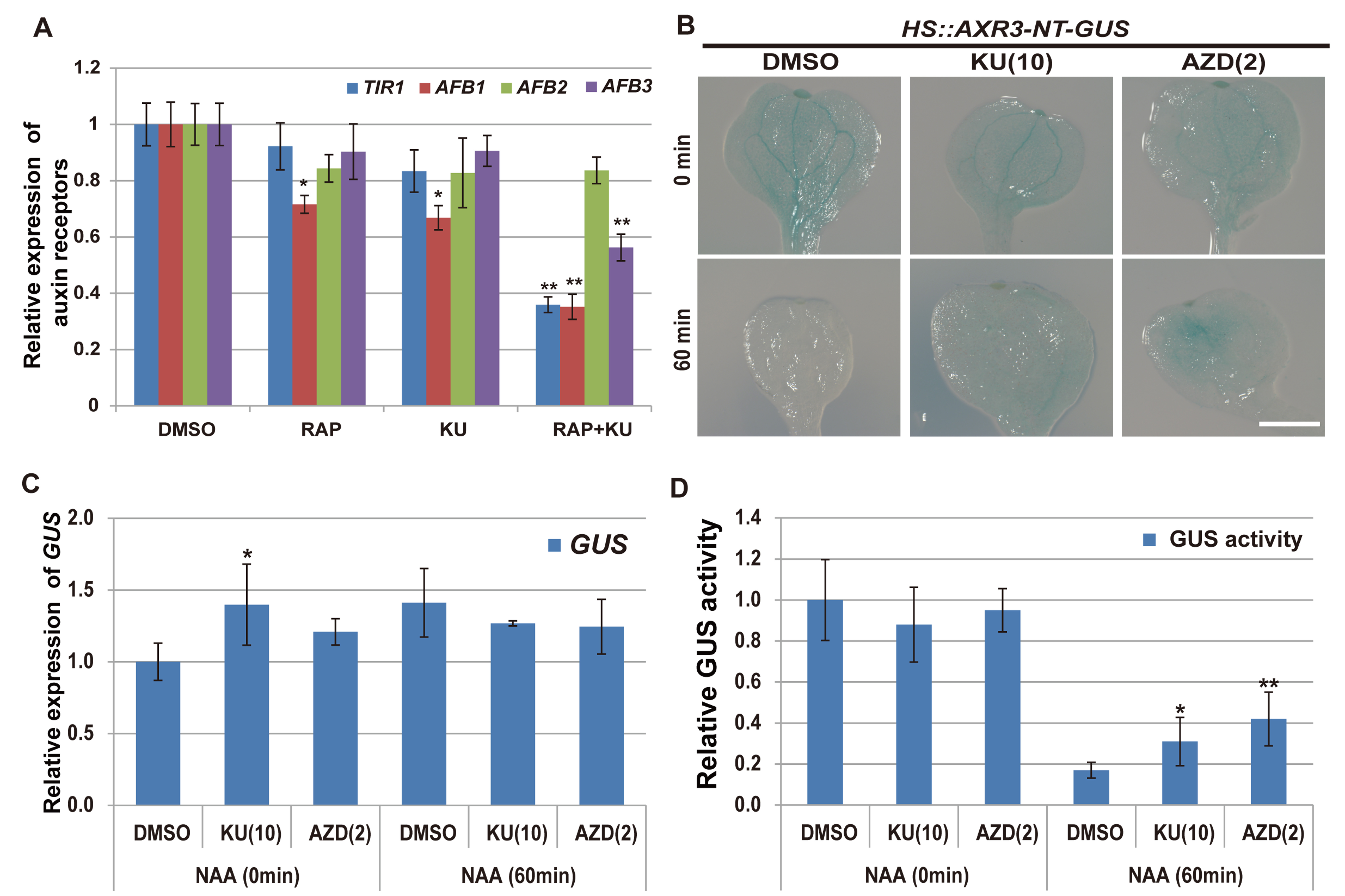

Supplement: Figure S6 — The expression of TIR1/AFBs and the degradation of AXR3NT-GUS under different TOR inhibitors treatment in Arabidopsis. (A) The expression of TIR1/AFBs during different TOR inhibitors treatment. Four days old BP12-2 seedlings grew in 0.5 MS medium with low light; then the root of seedlings were removed and stems were transferred to the medium containing TOR inhibitors [RAP (5 μM), KU (5 μM), RAP (5 μM) + KU (5 μM); DMSO was used as control] under normally growth condition for 48 h. (B) The GUS staining of HS:: AXR3NT-GUS under different TOR inhibitors treatment, KU(10 μM), AZD(2 μM), and DMSO was used as control, bar = 1 mm. (C,D) The relative expression of GUS and the relative activity of GUS in HS:: AXR3NT-GUS under different TOR inhibitors treatment; DMSO was used as control. Asterisks denote Student's t-test significance compared with control (*P < 0.05; **P < 0.01). Each value represents the mean ± SD of three independent experiments. [file Image6.JPEG]

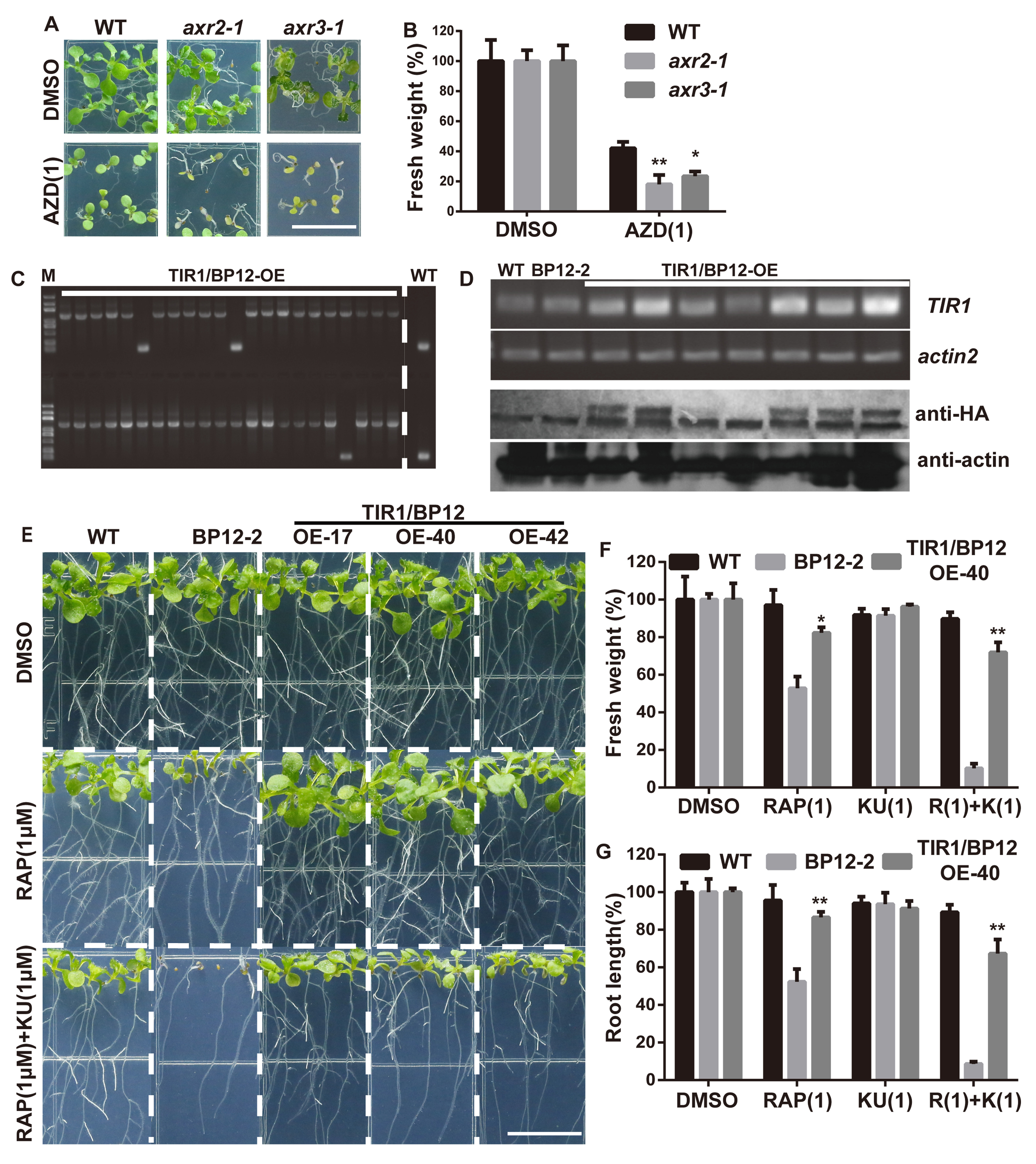

Supplement: Figure S7 — The auxin signal transduction was affect by TOR inhibitors in Arabidopsis. (A,B) The gain-of-function mutants axr2-1 and axr3-1 were sensitivity to asTORis compared to WT, bar = 1 cm. (C,D) The leaf PCR, semi-qPCR and western blot identification of TIR1/BP12-OE lines. (E) The phenotype of TIR1 /BP12 OE lines in medium with different TOR inhibitors, bar = 1 cm. (F,G) The fresh weight and lateral root numbers of WT, BP12-2 and TIR1-OE/BP12 lines under different TOR inhibitors treatment; DMSO was used as control. Asterisks denote Student's t-test significance compared with BP12-2 (*P < 0.05; **P < 0.01). Each value represents the mean ± SD of three independent experiments. [file Image7.JPEG]

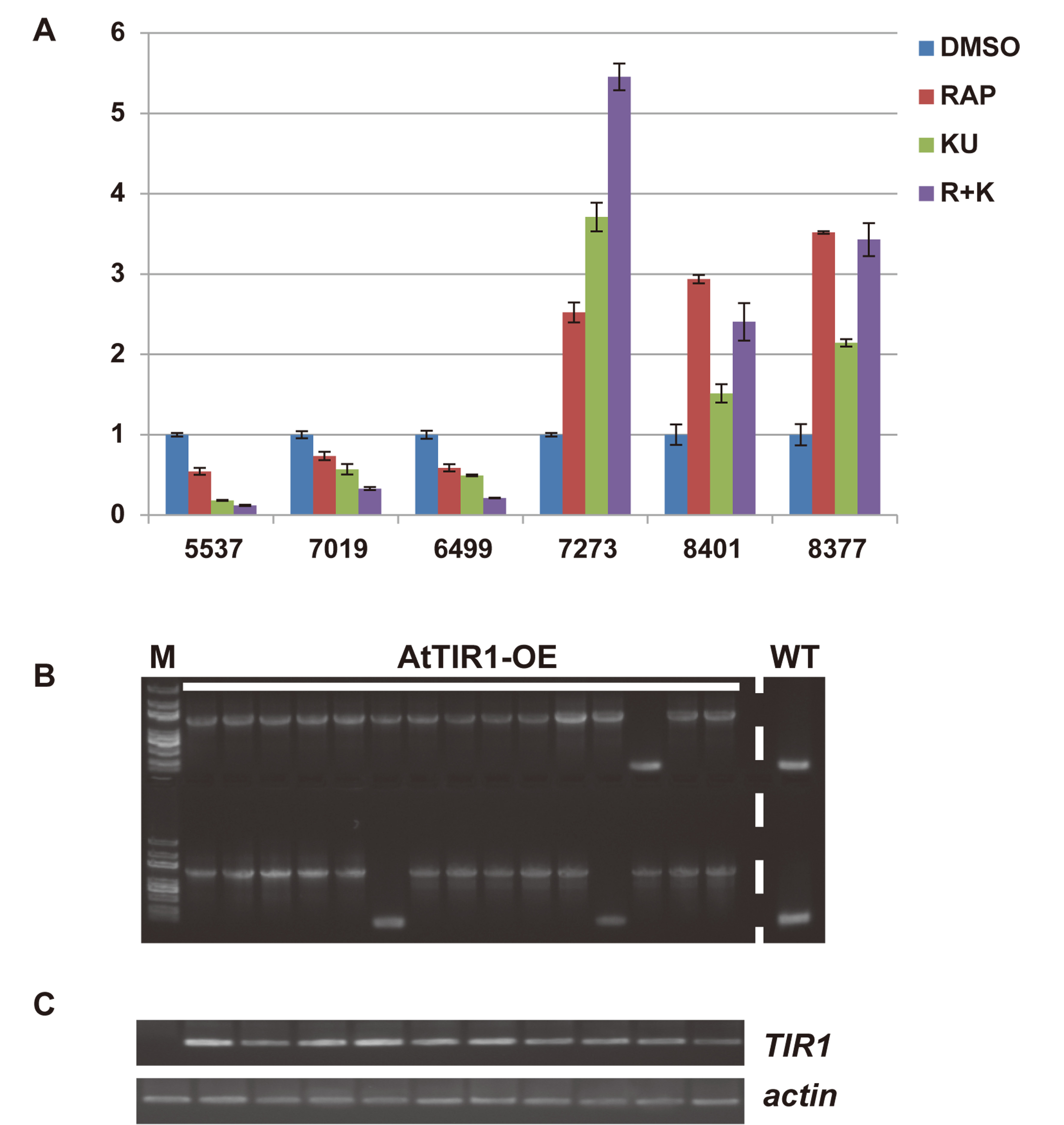

Supplement: Figure S8 — The validation of RNA-Seq data by qRT-PCR, and identification of potato TIR1-OE lines. (A) The validation of RNA-Seq data by qRT-PCR, and gene ID numbers are listed in Table S1. (B,C) The leaf PCR and semi-qPCR identification of potato transgenic lines of TIR1-OE. [file Image8.JPEG]

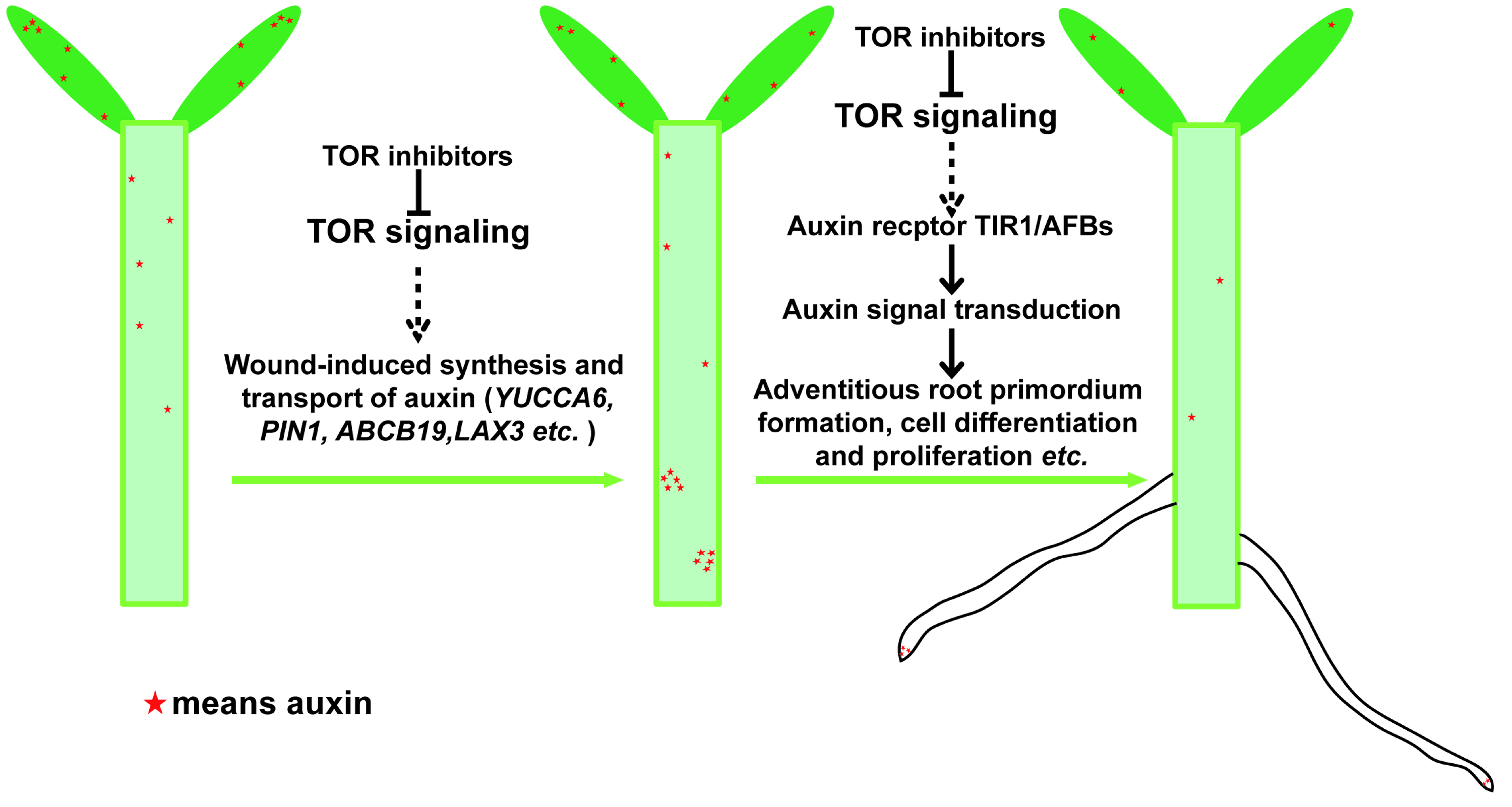

Supplement: Figure S9 — A model for the crosstalk between TOR signaling and auxin signaling during adventitious root formation. [file Image9.JPEG]
