# Supplementary material for: The TOR Pathway Is Involved in Adventitious Root Formation in Arabidopsis and Potato
Source: Front Plant Sci. 2017 May 12;8:784. doi: 10.3389/fpls.2017.00784 (PMC5427086; doi:10.3389/fpls.2017.00784)
Supplement: Table S3 — Summary of the sequence assembly after Illumina sequencing. [file Table3.DOC]

**Table S3 Summary of the sequence assembly after Illumina sequencing**

|  | Raw reads | Clean reads | Clean bases (Gb) | Error rate (%) | Q20(%) | Q30(%) | GC content (%) |
| --- | --- | --- | --- | --- | --- | --- | --- |
| DMSO | 54814750.67±9799560.55 | 53969582.00±9676586.56 | 8.10±1.45 | 0.01±0.00 | 97.27±0.08 | 93.08±0.17 | 42.86±0.07 |
| RAP | 47530034.00±642971.16 | 46721926.67±56335.79 | 7.01±0.09 | 0.02±0.00 | 96.34±0.62 | 90.92±1.38 | 42.75±0.16 |
| KU | 53581379.33±5993776.23 | 52735128.00±5894285.38 | 7.91±0.88 | 0.02±0.00 | 96.53±0.07 | 91.47±0.15 | 42.70±0.06 |
| RK | 60040264.00±11629156.71 | 59204390.67±11445419.39 | 8.88±1.72 | 0.02±0.00 | 96.86±0.07 | 92.04±0.17 | 42.65±0.06 |

**Gb: Giga base; Q20: percentage of bases with a Phred value of at least 20; Q30: percentage of bases with a Phred value of at least 30**

**The values represent the means of three times of repetition ± standard deviation**
