# Supplementary material for: The TOR Pathway Is Involved in Adventitious Root Formation in Arabidopsis and Potato
Source: Front Plant Sci. 2017 May 12;8:784. doi: 10.3389/fpls.2017.00784 (PMC5427086; doi:10.3389/fpls.2017.00784)
Supplement: Table S4 — Distribution of gene expression in the BP12-OE17 under the four different treatments. [file Table4.DOC]

| FPKM Interval | DMSO | rapamycin | KU | Rapamycin + KU |
| --- | --- | --- | --- | --- |
| 0~1 | 25015(54.12%) | 25068(54.24%) | 24958(54.00%) | 25126(54.37%) |
| 1~3 | 4440(9.61%) | 4432(9.59%) | 4436(9.60%) | 4347(9.41%) |
| 3~15 | 8519(18.43%) | 8401(18.17%) | 8428(18.24%) | 8317(18.00%) |
| 15~60 | 5995(12.97%) | 5984(12.95%) | 6079(13.15%) | 6096(13.19%) |
| >60 | 2248(4.90%) | 2330(5.04%) | 2314(5.01%) | 2330(5.04%) |

**Table S4 Distribution of gene expression in the BP12-OE17 under the four treatment**

**FPKM:** **Expected number of fragments per kilobase of transcript sequencee per millions base pairs sequenced. Ratios of gene number to total gene number are presented in parentheses**
